# Large‐Scale Flat Silk Cocoons as a Highly Effective Salt‐Resistant Low‐Cost Solar‐Powered Evaporator

**DOI:** 10.1002/advs.202511284

**Published:** 2025-09-15

**Authors:** Tiancheng Wei, Qichao Cheng, Leihao Lu, Quan Wan, Qi Wu, Ruixi Shao, Zongpu Xu, Jie Wang, Yajun Shuai, Chuanbin Mao, Mingying Yang

**Affiliations:** ^1^ Key Laboratory of Silkworm and Bee Resource Utilization and Innovation of Zhejiang Province Institute of Applied Bioresource Research College of Animal Science Zhejiang University Hangzhou Zhejiang 310058 P. R. China; ^2^ Department of Biomedical Engineering The Chinese University of Hong Kong Sha Tin P. R. China

**Keywords:** flat silk cocoon, janus, salt resistance, solar evaporator

## Abstract

Solar seawater evaporators are a promising sustainable solution for freshwater production, yet they face challenges related to efficiency, evaporation rate, salt resistance, and the large‐scale manufacturing of evaporative materials. Here, we demonstrate that flat silk cocoons (FSC), produced in large sizes (70 cm × 200 cm) by utilizing silkworms to spin silk fibers on flat surfaces, serve as evaporators with remarkable performance in salt resistance and evaporation. Specifically, the FSC s modified into a Janus‐structured evaporator (FJE) by coating the top (thin) portion with superhydrophobic polydimethylsiloxane and the bottom (thick) portion with superhydrophilic polypyrrole. The Janus structure enables the FJE to transport water to the superhydrophilic/hydrophobic interface through its bottom portion and evaporate without salt precipitation. Simultaneously, the top portion absorbs sunlight and transfers heat to the bottom portion within the evaporator, promoting evaporation while minimizing heat loss. The FJE demonstrates exceptional cost‐effectiveness (258.2 g h^−1^ USD^−1^), high evaporation rate (3.075 kg m^2^ h^−1^), and an impressive evaporation efficiency (79.73%). Notably, the superhydrophilic layer of the Janus structure enhances water exchange, enabling sustained evaporation for 18 h in 20 wt.% saline solutions without salt deposition under sunlight. The FJE represents a new generation of eco‐friendly, cost‐effective, salt‐resistant, and thermo‐localized evaporators.

## Graphical abstract

This work is the first large‐scale preparation of solar seawater evaporators using natural flat silk cocoons. The flat silk cocoon's natural fibrous transverse multilayers combined with a Janus structure minimize heat loss and salt crystallization, while providing extremely fast water transport and evaporation rates. Easy and economical large‐scale evaporators solve the challenge of transverse hierarchical evaporators and optimize evaporation economics.

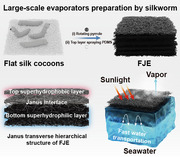

## 1 Introduction

The escalating issue of freshwater scarcity has elevated the significance of research in seawater desalination using solar seawater evaporators.^[^ ^1^ ^]^ Conventional solar evaporators prioritize high evaporation rates.^[^ ^2^ ^]^ However, prolonged evaporation leads to salt crystal accumulation on the surface, obstructing sunlight absorption and consequently reducing the evaporation rate.^[^ ^3^ ^]^ The Janus evaporator, distinguished by its hydrophobic and hydrophilic layers, addresses this challenge.^[^ ^4^ ^]^ The hydrophobic layer facilitates solar absorption to generate heat, while the hydrophilic layer aids in water transportation, mitigating heat loss and crystallization risks by avoiding direct contact between the evaporating layer and the water column.^[^ ^4^, ^5^ ^]^

Nevertheless, salt crystal formation at the hydrophobic‐hydrophilic interface can still impede the evaporation rate and long‐term salt resistance of the Janus evaporator.^[^ ^6^ ^]^ Combining a transverse hierarchical structure with the Janus design further diminishes heat loss and crystallization risks.^[^ ^7^ ^]^ However, the current fabrication methods predominantly rely on 3D printing,^[^ ^8^ ^]^ ice crystal templating, ^[^ ^7^, ^9^ ^]^ and electrospinning. These methods have the cost and safety limitations of using high voltage electricity and special equipment, and the mechanical strength and durability of the fibers prepared by electrospinning are completely inferior to that of natural silk fibres, lacking a sustainable and straightforward approach for large‐scale production of transverse hierarchical Janus structure evaporators using environmentally friendly materials.^[^ ^3^, ^10^ ^]^

The flat silk cocoons (FSC), a natural silk derivative, represent a distinctive 3D layered porous material characterized by stacking 2D fiber networks on flat plates through continuous silk spinning by silkworms.^[^ ^11^ ^]^ Notably, FSC offers advantages in terms of accessibility, cost‐effectiveness, and scalable production, owing to their inherently porous transverse layered structures.^[^ ^12^ ^]^ While existing research on FSC spans mechanical reinforcement,^[^ ^11c^ ^]^ medical applications such as dressings,^[^ ^13^ ^]^ and sensing technologies,^[^ ^14^ ^]^ investigations into their environmental applications remain scarce.^[^ ^15^ ^]^ The loose, layered, and porous architecture of FSC makes them conducive to efficient water transport, while their natural transverse hierarchical structure contributes to thermal resistance.^[^ ^2^, ^7^, ^16^ ^]^ The silkworm has been reared and utilized in China for about 3000–4500 years, where the production of cocoons reaches 8 00 000 tons per year. FSC is a kind of silkworm cocoon that is green and pollution‐free.^[^ ^17^ ^]^ Compared to silk fabrics, which require complex processes and high costs such as degumming, reeling, and weaving, low‐cost FSC can be easily obtained simply by silkworms spinning silk on a flat surface. Industrialized silkworm farming can produce FSC in large quantities at any time. These attributes, coupled with their affordability and accessibility, render FSC highly practical and cost‐effective to be applied as transverse layered Janus evaporators. Compared with other low‐cost fibers, FSC has extremely high environmental stability and can be easily obtained in large quantities, giving it a unique advantage in the industrialization of seawater evaporators in the future.

This study presents the first attempt at developing a transverse hierarchical Janus seawater evaporator fabricated by direct modification of FSC. The process involved in situ polymerization of superhydrophilic polypyrrole (PPy) on the constituent fibers throughout FSC. Subsequently, polydimethylsiloxane (PDMS) was sprayed and cured on the thin top layer of FSC to form a superhydrophobic layer on the top of FSC, thereby establishing a transversal hierarchical Janus structure. The resultant structure is an FSC‐based Janus‐structured solar evaporator (FJE) with low cost, high evaporation efficiency, and strong salt resistance, as confirmed by a combination of experimental and modeling studies. The fibrous superhydrophilic bottom portion of the FJC allows the water to be efficiently delivered from brine to the boundary between the superhydrophilic and superhydrophobic portions and rapidly evaporates from there without salt precipitation. The evaporated water vapor can be collected to produce water that meets the standard of the World Health Organization (WHO). In the meantime, the fibrous superhydrophobic top portion of the FJE effectively absorbs solar energy and transports the heat downward to promote evaporation. Overall, the unique fibrous Janus structure of the FJE allows the solar energy to be localized inside the evaporator to prevent energy loss and increase energy utilization efficiency.

## 2 Results and Discussion

### 2.1 Synthesis and Structure of FJE

**Figure 1:**
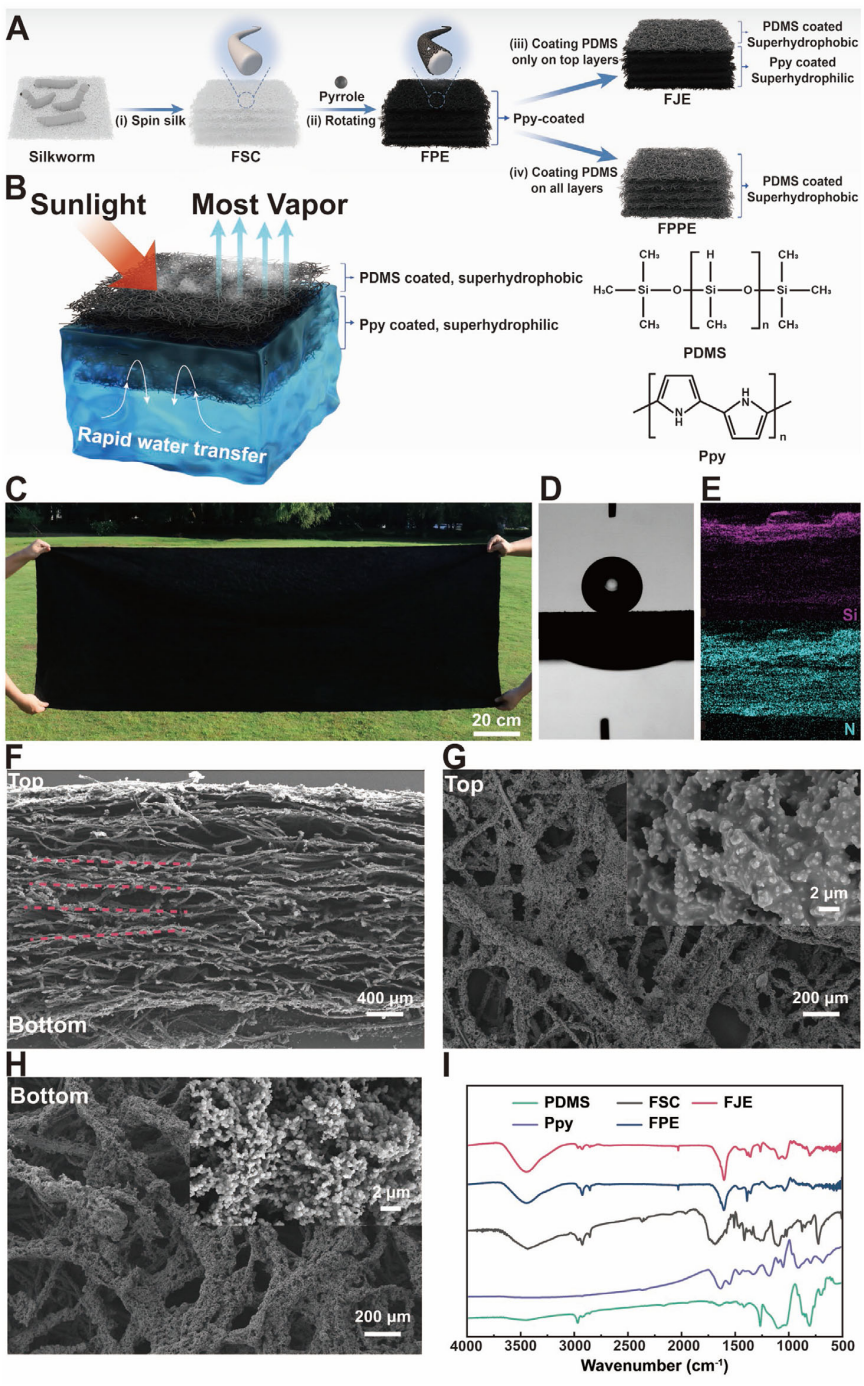
Fabrication and characterization of FJE. A) Method for formulating FJE and evaporators of different components. (i). Silkworms spit silk directly on a flat surface to form a 2D film; the 2D film is stacked to form a 3D flat silk cocoon. (ii). The FSCs are immersed in a continuous vibration environment where polypyrrole grows in situ on the surface of all the fibers, and the FPE is formed by super‐hydrophilic Ppy modification. (iii). Only the top layer (thin) of the FPE is sprayed with PDMS, and the FJE is formed by curing. (iv). All the fibers of the FPE are sprayed with PDMS, and the FPPE is formed by curing. B) The FJE has a Janus structure. Schematic diagram of efficient evaporation and salt resistance mechanism of FJE. C) Photographs of FJE with large‐scale production (70 × 200 cm). D) The water contact angle between the top and bottom of FJE. E) Elemental distributions of N and Si of the cross‐section of FJE by EDS analysis. (F‐H) SEM photographs of F) cross‐section, G) top, and H) bottom of FJE; the inset is the enlarged SEM photograph. The red dashed lines in (F) indicate the hierarchical structure of FJE. I) FTIR analysis results of FJE and its components.

The FSC were prepared in a way differently from round cocoons by silkworms. To fabricate FSC, the silk fibers were directly spun by the silkworm on the plain platform compared to natural round cocoons (Video S1, Supporting Information).^[^ ^11a,c^ ^]^ The fabrication process of the evaporator is depicted in **Figure** **1**A. PPy was first grown on the fibers throughout the FSC, and the top surface of the FSC was then coated with PDMS using a spray gun, producing FJE with the bottom and top surface being hydrophilic and hydrophobic, respectively. As controls, the FSC that was fully coated with Ppy throughout acted as a completely hydrophilic evaporator termed FPE, and FP E was further fully coated with PDMS to form a completely hydrophobic FSC‐based PDMS‐coated evaporator termed FPPE. Different compositions led to different evaporation rates of FJE, FPE, and FPPE, with FJE showing the most vapor production (Figure 1B; Figures S1 and S2, Supporting Information). Because FSC could be virtually formed in any size, the preparation of large‐size evaporators is simple and feasible (Figure 1C; Figure S3, Supporting Information). In this experiment, our demonstration was conducted on a 4 × 4 cm FSC.

The difference in thickness between FSC and FJE is ≈4 mm, leading to a color change from white to black after modification (Figure S4, Supporting Information). The top and bottom portions of FJE yield a water contact angle (WCA) of 153.6° and 0°, which indicates FJE's Janus structures (Figure 1D). The Si element distribution (Figure 1E) indicates that PDMS is predominantly concentrated in the top (20%) region of FJE. Further detailed insights into the elemental distribution are provided in Figures S5–S7 (Supporting Information). The transverse hierarchical arrangement of FJE was manifested as the stacking of many layers (Figure 1F). The top and bottom portions of FJE exhibited porous configurations composed of randomly distributed fibers (Figure 1G,H). PPy growth in situ on the silk fibers increased the surface roughness in FPE. A thin layer of PDMS was observed adhering to the PPy coating on the top of FSC, as revealed by comparing the top and bottom SEM images of FJE.

The FTIR analysis (Figure 1I) of FJE revealed characteristic peaks at 1647 and 1550 cm^−1^, attributing to amide I and amide II of silk proteins, respectively.^[^ ^18^ ^]^ Additionally, a peak at 1166 cm^−1^ was observed, indicating the presence of C‐N bonds and confirming the in situ growth of PPy.^[^ ^7b^ ^]^ Furthermore, a peak at 1095 cm^−1^ verified the incorporation of Si‐O‐Si bonds, signifying the presence of PDMS in the evaporator.^[^ ^19^ ^]^ SEM imaging displayed the cross‐section, top, and bottom views of FPE and FPPE, revealing the retention of the transverse hierarchical structure following the in situ growth of PPy and PDMS on the evaporator (Figure S8, Supporting Information). However, the WCA indicated that increasing the number of PDMS spraying cycles led to a thicker hydrophobic layer, ultimately resulting in the loss of Janus properties. Thus, PDMS spraying should be limited to a maximum of three times. It can be concluded from the above results that PPy and PDMS have been coated on FSC to form transverse hierarchical Janus structures in FJE.

### 2.2 Janus Properties, Solar Absorption, and Mechanical Stability of FJE

**Figure 2:**
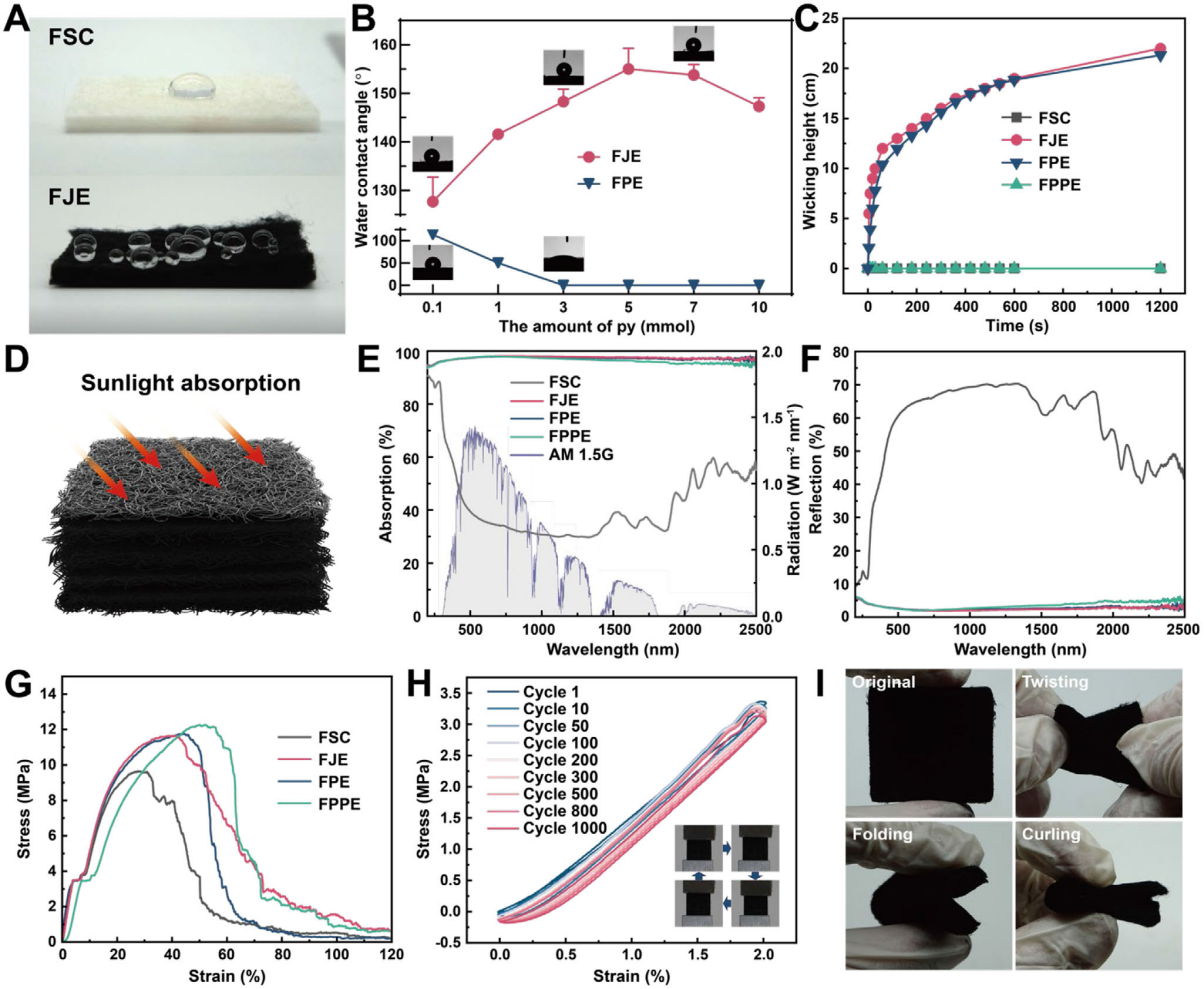
Optical, mechanical, and absorption properties of different evaporators. A) Photographs of water droplets on the surface of FSC versus FJE. B) WCAs of the top surfaces of FJE, FPE with different PPy contents, and the insets are photographs of the WCA. C) Water absorption rates of FSC, FJE, FPE, and FPPE. D) Schematic of sunlight absorption of FJE evaporator. E,F) the sunlight absorption (E) and reflection (F) of FSC, FJE, FPE, and FPPE. G) Mechanical strength of FSC, FJE, FPE, and FPPE in breaking tensile. H) Mechanical strength variation of FJE for 1000 cycles of tension: the inset is a photograph of one cycle of the cyclic tension. I) Photographs of FJE being folded, twisted, and curled.

FSC exhibited hydrophobicity due to its fluffy and porous properties (**Figure** **2**A), resulting in the presence of an air layer between the FSC surface and the droplets, while the droplets were suspended on the surface of FJE due to the superhydrophobicity of its surface. PPy became hydrophilic after p‐Toluenesulfonic acid sodium (PTS) doping (Figure 2B). As the amount of synthesized hydrophilic PPy increased, surface roughness on the micro‐nano scale emerged on the silk fibers (Figure S9, Supporting Information). This caused evaporators to change from hydrophobic to superhydrophilic and exhibit faster water absorption (Video S2, Supporting Information). Spherical water droplets stayed on the top hydrophobic portion of FJE, confirming its superhydrophobic nature. As shown in Figure S10 (Supporting Information), FSC, FPE, and FJE had an air permeability of 1151.28, 908.24, and 905.35 mm s^−1^, respectively. Despite the increased amount of PPy led to decreased air permeability, pore diameter, and porosity, FJE maintained relatively high air permeability, pore diameter, and porosity compared to FSC. The water absorption properties of FSC were altered after modification. FSC and FPPE were essentially nonabsorbent due to their inherent hydrophobic characteristics (Figure 2C). The average water absorption rate of FPE in the first 60 s was 2 mm s^−1^, similar to FJE. The superior water absorption capacity of the superhydrophilic layer on the bottom surface and the presence of a superhydrophobic layer on the top surface render FJE particularly well‐suited for deployment as a seawater evaporator (Video S3, Supporting Information).

FJE achieved evaporation through optical energy absorption and its subsequent conversion into thermal energy (Figure 2D). FSC exhibited an average optical absorption rate of 50%. In contrast, the optical absorption rate of FJE was 98%, similar to FPE (98.1%) and FPPE (96.3%) (Figure 2E,F). Additionally, the absorption capacity of the material showed a slight increase with the escalation of PPy synthesis (Figure S11, Supporting Information). Conversely, there was a marginal decrease in absorption as the number of PDMS spraying cycles increased. This decline in absorption can be caused by the effect of PDMS on reducing surface roughness, resulting in partial light reflection.^[^ ^18^, ^20^ ^]^ These findings corroborate that augmenting PPy content while reducing PDMS spraying cycles enhances the evaporator's light absorption capability. Moreover, the exceptional light absorption rate of FJE positions is desired for producing solar evaporators.

The mechanical strength of FJE and other groups was evaluated by a tensile‐breaking test. FSC exhibited a breaking strength of 9.65 MPa at 31% tensile strain. Conversely, FJE presented a breaking strength of 11.62 MPa at 42% tensile strain, showcasing an increase in tensile strain and breaking strengths attributed to the additional strength provided by the PPy and PDMS composite coating on the fibers. The results of FPE and FPPE similarly validate this point, with a breaking strength of 11,83 MPa at 44% tensile strain and a breaking strength of 12.12 MPa at 47% tensile strain, respectively (Figure 2G; Figure S12, Supporting Information). The elongation at break was greater for FJE, with a strain of 41% at the beginning of breaking and 109% at complete break. This behavior can be attributed to the nondirectional fiber composition of FSC, because randomly oriented fibers do not break simultaneously. The uniform mechanical properties of different FSCs arise from the silkworm's consistent lateral head movements during spinning, which actively pull silk from the spigot in a figure‐eight pattern.^[^ ^21^ ^]^ Since sericin is a layer on the silk fibroin fibre, Ppy will be sandwiched between the PDMS and sericin layers and glued by these layers, increasing the strength and toughness of the FJE material.^[^ ^11c^ ^]^ The mechanical strength of FJE remained unchanged even after 1000 cycles of tensile stretching (Figure 2H). Moreover, under 20% elongation, both FSC and FJE retained robust mechanical strength following 1000 cycles of tensile stretching (Figure S12, Supporting Information). FJE was resistant to folding, twisting, and curling, indicating its remarkably high toughness (Figure 2I). Consequently, the combination of its high mechanical strength (11.62 MPa), elongation at break (109%), and the unaltered mechanical strength after 1000 cycles underscores the exceptional mechanical stability of FJE.

### 2.3 Warming and Evaporation Characteristics of FJE

**Figure 3:**
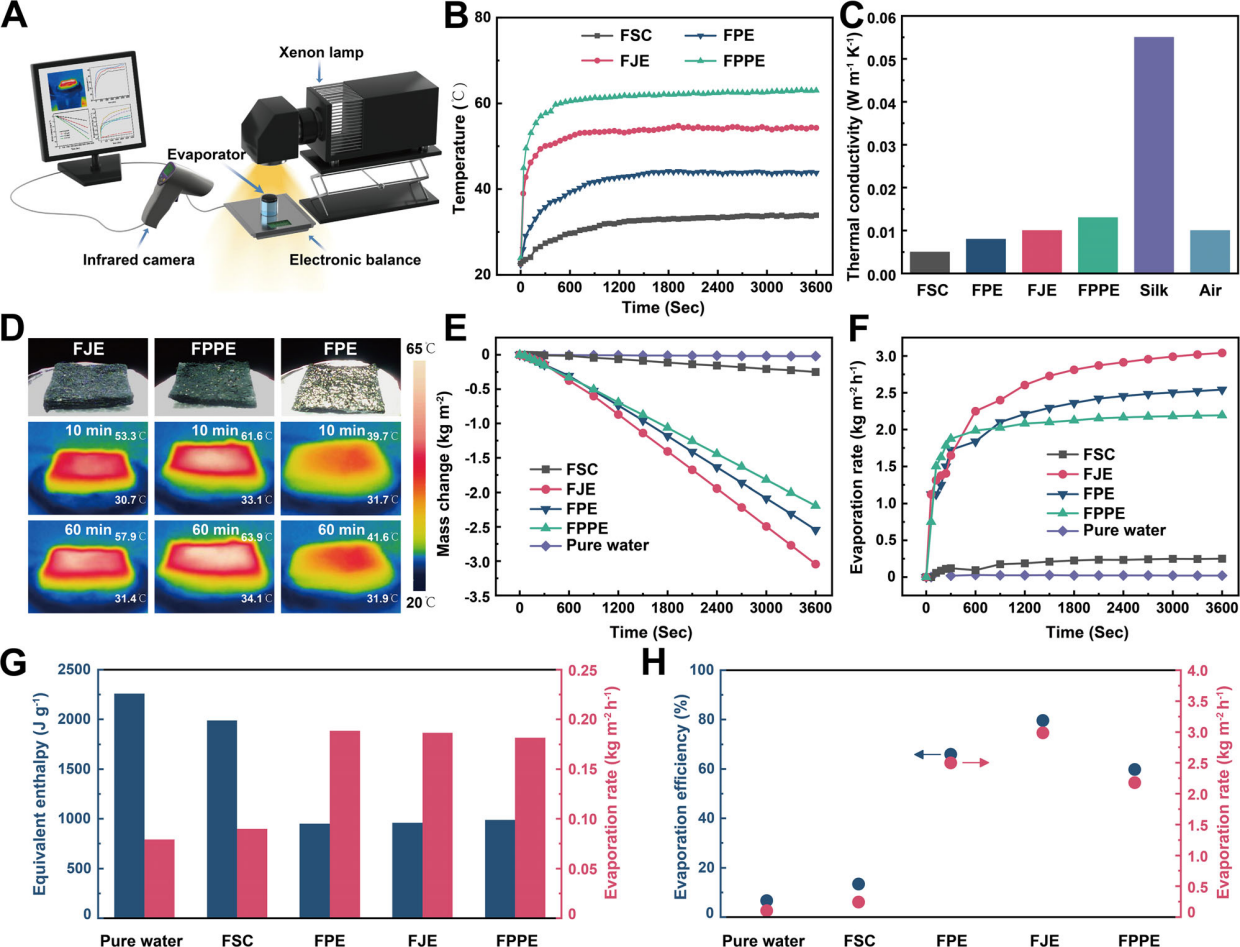
Performance of the saltwater evaporation of our FJE evaporator. A) Schematic of the laboratory test of saltwater evaporation. B) Warming of FSC, FJE, FPE, FPPE in water under sunlight. C) Longitudinal thermal conductivity of FJE and other groups at 40 °C. D) Thermal photographs for the top and bottom temperatures of FSC, FJE, and FPPE under one sunlight. Comparison of E) water mass changes and F) evaporation rates between FJP and other groups. G) Evaporation rate and equivalent enthalpy of water in a dark environment (25 °C) for FJE and other groups. H) Comparison of evaporation efficiency and evaporation rate for FJE and other groups.

Initially, the laboratory setup (**Figure** **3**A) was established, followed by brine evaporation. The evaporator was positioned atop a water buoyed by polystyrene foam and connected to the water via filter paper.^[^ ^6b^ ^]^ After an hour of solar irradiation, temperature readings were taken, revealing an average surface temperature of 33.6 °C for FSC, while FJE, FPE, and FPPE exhibited temperatures of 54.8, 44.3, and 63.2 °C, respectively (Figure 3B). The escalation in evaporation temperature resulted in a maximum temperature difference of 29.6 °C between FJE and FPPE, attributable to a denser superhydrophobic layer of FPPE (Figure S13, Supporting Information). Notably, while silk fiber displayed a considerably high thermal conductivity (Figure 3C; Figure S14, Supporting Information) at 0.055 W m^−1^ k^−1^, FSC exhibited the longitudinal thermal conductivity of merely 0.005 W m^−1^ k^−1^, a tenth of silk fiber's conductivity, owing to its multilayered fluffy structure. FJE exhibited a modest thermal conductivity of 0.01 W m^−1^ k^−1^ at 40 °C, owing to the heat‐blocking effect of its transverse hierarchical structure.^[^ ^16^ ^]^ Furthermore, Figure 3D depicts the temperature distribution across the evaporator's cross‐section, revealing a temperature difference of 26.5 °C between the top and bottom of FJE following a single instance of solar irradiation and wetting. Conversely, FPE demonstrated a temperature disparity of 9.7 °C between its top and bottom temperatures, owing to its complete hydrophilicity (and thus rapid energy absorption by water) and lack of a Janus structure. FPPE, characterized by an utterly hydrophobic structure with a transverse hierarchical arrangement, displayed a temperature difference of 29.8 °C. However, its excessive thickness led to significant heat dissipation through thermal radiation, yielding a lower bottom layer temperature of 34.1 °C.

Indoor simulated tests revealed negligible evaporation rates for pure water and FSC. However, FPE, FJE, and FPPE displayed high evaporation rates of 2.54, 3.08, and 2.19 kg m^−2^ h^−1^ (Figure 3E,F). Evaporation rates increased and then decreased with the increment in PDMS spraying, attributed to the thermal blocking effect of the hydrophobic transverse hierarchical structure. Similarly, as the amount of PPy increased, the evaporation rate followed a similar pattern due to the decrease in porosity, specific surface area, and permeability because these structural changes hinder vapor escape (Figure S10,S11, and S15, Supporting Information). The evaporation rate of FJE linearly correlated with light intensity, confirming its photostability (Figure S16, Supporting Information). The enthalpy decreased from 2260 J g^−1^ for pure water to 961.15 J g^−1^ for FJE because water adsorption by the porous fiber layer structure in FJE enhances water dispersion, reducing the number of hydrogen bonds in water clusters on the fiber surface and the energy required for water molecules to evaporate, also the DSC results showed the same trend^[^ ^2^, ^7^ ^]^ (Figure 3G; Figure S17, Supporting Information). FJE exhibited the highest evaporation efficiency at 79.73%, surpassing FPE (66.05%) and FPPE (59.84%) (Figure 3H; Figure S17, Supporting Information). Comparison of heat loss pathways among FPE, FJE, and FPPE (Figure S18 and Table S1, Supporting Information) revealed the superior heat‐blocking capabilities of FJE.

### 2.4 Salt Resistance of FJE

**Figure 4:**
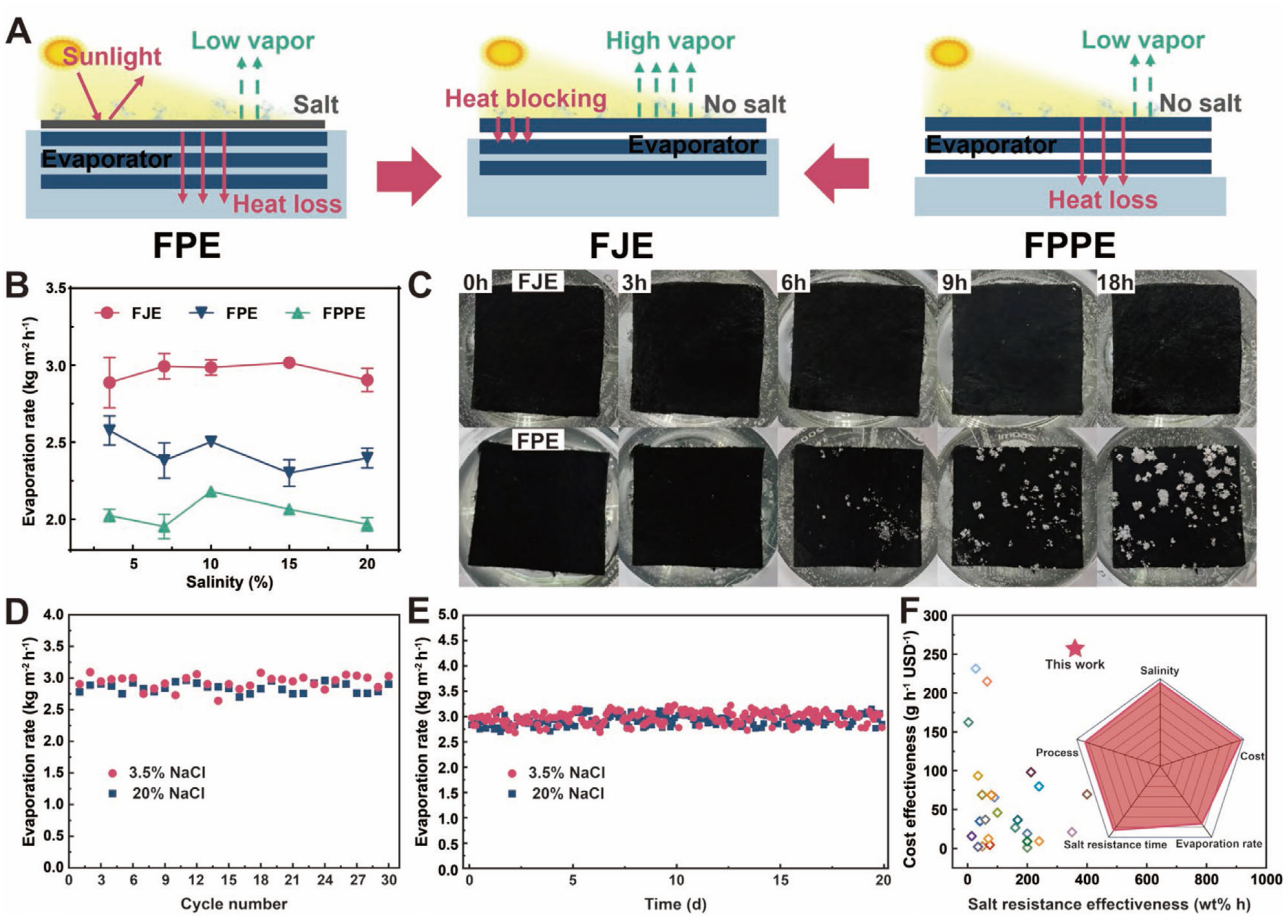
Indoor performance test of our FJE evaporators. A) Schematic of FJE's salt resistance mechanism. B) Evaporation rates of FJE, FPE, and FPPE at different salt concentrations, C) Photographs of continuous evaporation of FJE and FPE at 20 wt.% salt concentration, D) Evaporation rates of FJE cycle‐washed evaporation for 30 times at 3.5 and 20 wt.% salt concentrations, E) evaporation rate of FJE at 3.5% and 20% salt concentrations for 18 h per day for 20 d, F) Comparison of FJE with recently published studies on cost‐effectiveness & salt‐resistant evaporators. The inset shows the superiority of the FJE in terms of salt‐resistance concentration, salt‐resistance time, cost, process time, and evaporation rate.

The sustained salt resistance and efficient evaporation performance of FJE primarily stemmed from its Janus structure (**Figure** **4**A). The elevated temperature of the hydrophobic layer expedited brine evaporation, while the superhydrophilic layer facilitated rapid water exchange, ensuring that salt concentration at the evaporation interface remained below the crystallization threshold (Figure 1B; Figures S1 and S2, Supporting Information).^[^ ^5^, ^6^, ^22^ ^]^ In contrast, the uniform heating of the all‐hydrophilic FPE led to inefficient water transportation at the top layer, resulting in salt crystallization and reduced evaporation rates.^[^ ^3^, ^23^ ^]^ Although FPPE, being entirely hydrophobic, prevents salt crystallization, its lower bottom layer temperature hampers evaporation efficiency.^[^ ^24^ ^]^ FJE maintained an average evaporation rate of 2.93 kg m^−2^ h^−1^ in 20 wt.% brine to validate the evaporator's resistance to high brine concentrations. In contrast, FPE and FPPE exhibited lower evaporation rates (average 2.43 and 2.03 kg m^−2^ h^−1^, respectively) due to increased heat loss (Figure 4B; Table S1, Supporting Information). Comparative analysis of evaporators with varying PPy contents yielded similar results (Figure S19, Supporting Information). Additionally, Figure 4C illustrates the continuous evaporation of FPE and FJE in 20 wt.% brine, facilitated by polyethylene foam suspension and filter paper for water supply. While FPE manifested salt crystal formation after 6 h, FJE showed minimal crystallization after 18 h (Figure S20, Supporting Information). SEM images verified no salt deposition after 18 h of continuous evaporation at the bottom of FJE (Figure S19, Supporting Information). Si and Na elemental signals of cross sections show the transverse hierarchical structure of FJE with salt resistance (Figures S21 and S22, Supporting Information). The range of Si and Na elements shows a complementary state, indicating the absence of salt crystallization in the hydrophobic layer. Meanwhile, the Na element is present in multiple peaks, proving the transverse hierarchical structure.

To assess the evaporation stability of FJE, it was tested by 30 wash cycles, with each cycle comprising 9 h of evaporation, in both 3.5 and 20 wt.% brine. The sample exhibited no declining trend, maintaining average evaporation rates of 2.94 and 2.85 kg m^−2^ h^−1^, respectively (Figure 4D). Similarly, during 20 days with daily 9‐h evaporation cycles, FJE exhibited consistent evaporation rates of 2.98 and 2.93 kg m^−2^ h^−1^ in 3.5 and 20 wt.% brine, respectively. These results validated the sustained stability of this sample (Figure 4E). Furthermore, FJE maintained a high evaporation rate (the same as the brine environment) under sustained extreme liquid environments, including 0.1 m NaOH, 0.1 m HCl, and ultrasonic conditions for 10 h. The amino acid sequence of “GAGAGS” accounts for the majority of silk fibres. Silk proteins can only be dissolved by prolonged immersion in strong acids and bases, and the evaporation efficiency remains stable in different environments (Figure S23, Supporting Information). Such data affirm the robust stability of FJE.

Compared to other reported evaporators, our salt‐resistant and cost‐effective FJE evaporators have outperformed them (Figure 4F; Table S2, Supporting Information), considering factors such as cost, salt‐resistant concentration, duration of salt resistance, evaporation rate, and process time. While the evaporation efficiency of FJE is average compared to other studies, FJE has a low cost of 11.91 $ m^−2^ (Table S3, Supporting Information) and exhibiting a salt resistance of 20 wt.% and a saline resistance duration of 18 h, alongside a synthetic process time of 3 h and an evaporation rate of 3.075 kg m^−2^ h^−1^ under sunlight. The inset in Figure 4F illustrates the significant superiority of FJE across all five metrics. Two parameters were introduced to better illustrate the cost‐effectiveness and salt‐resistant properties of FJE. The first, cost‐effectiveness (g h^−1^ USD^−1^), denotes the weight (in grams) of pure water obtained through evaporation in 1 h per $1 spent, showcasing the evaporator's cost‐effectiveness. The second, salt resistance effectiveness (wt.% h), combines salt resistance concentration (wt.%) and salt resistance time (hours), indicating both the salt concentration requirement and the duration of continuous salt resistance, providing a more comprehensive assessment of pragmatic salt resistance (Figure 4F). For instance, while the reported KGM/PVA/Fe‐MOF hydrogel demonstrates a cost‐effectiveness of 214.7 g h^−1^ USD^−1^, its salt resistance effectiveness is merely 66 wt.% h.^[^ ^25^ ^]^ Conversely, the reported CNTs@SiO_2_ Nanofibrous Aerogels exhibit a cost‐effectiveness of only 19.23 g h^−1^ USD^−1^ but boast a high salt resistance effectiveness of 200 wt.% h.^[^ ^3e^ ^]^ In contrast, our FJE demonstrates remarkable cost‐effectiveness at 258.2 g h^−1^ USD^−1^ and an impressive salt resistance effectiveness of 360 wt.% h. Consequently, our FJE is highly cost‐effective and salt‐resistant.

### 2.5 Outdoor Water Collection Capacity of FJE

**Figure 5:**
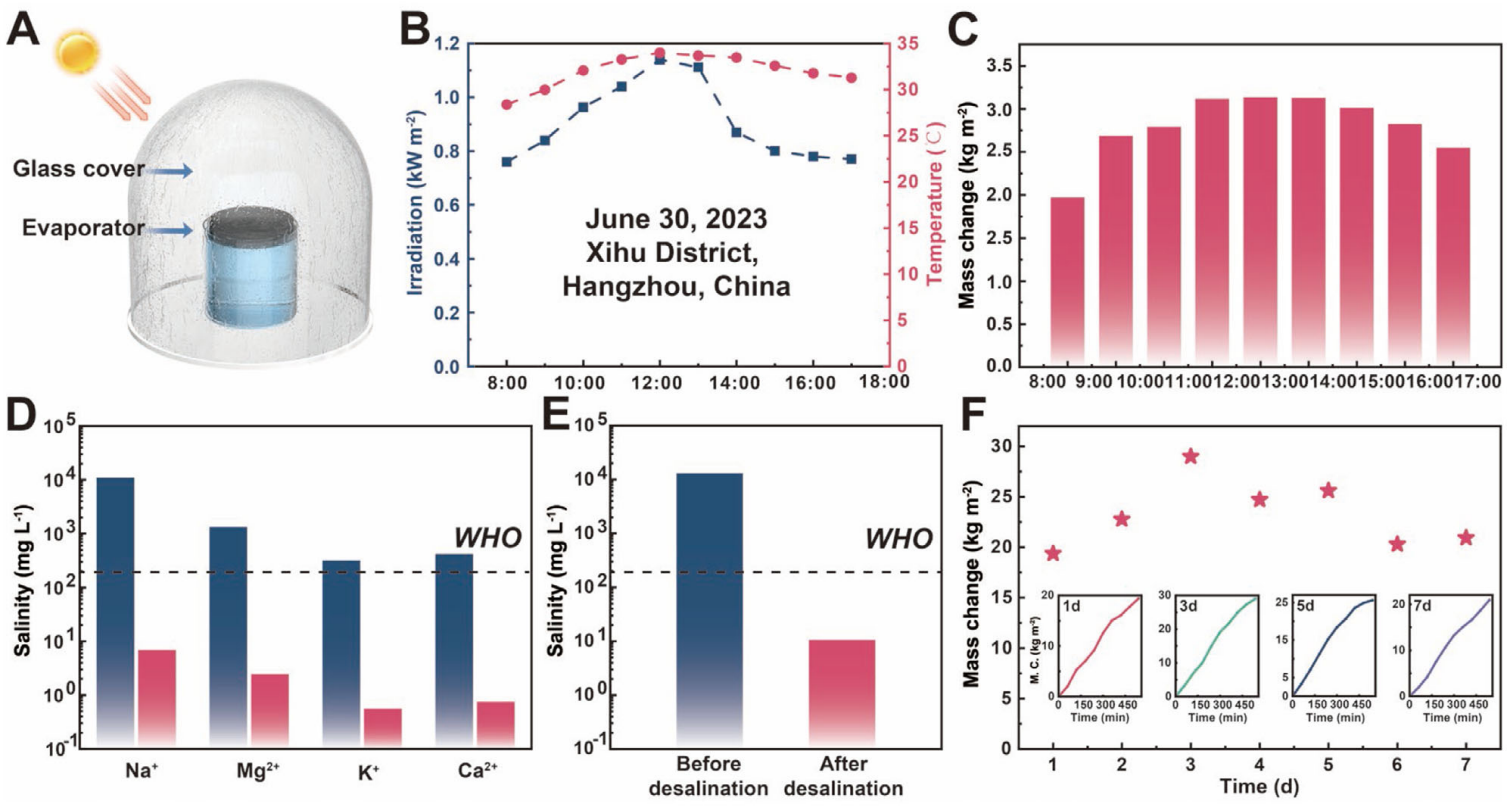
Outdoor performance test of FJE. A) Schematic of outdoor evaporation, B) sunlight and temperature variations during a day of outdoor evaporation, specific information: June 30, 2023, Xihu District, Zhejiang, China, C) weight of water collected per hour during a day of outdoor evaporation, D) ionic concentration of water collected by outdoor evaporation versus the water before evaporation, E) total ionic concentration of the water before and after outdoor evaporation, and F) weight of water collected by outdoor evaporation per day during a week, with the insets showing the evaporation rates for days 1, 3, 5, and 7.

The customized circular evaporation equipment was utilized to assess the outdoor water collection capacity of FJE, featuring the placement of the evaporator on the internal water sink, with the outer glass shell utilized for collecting steam condensation^[^ ^26^ ^]^ (**Figure** **5**A; Figure S24, Supporting Information). The fluctuations in sunlight intensity and temperature throughout a day of outdoor evaporation (8:00–17:00, totaling 9 h) conducted with FJE are analyzed (Figure 5B) by applying simulated seawater. This experiment started on June 30, 2023, in the Xihu District, Hangzhou, China. Throughout the 9‐h duration, the average solar intensity was recorded at 0.91 kW m^−2^, with the average temperature at 32.07 °C and humidity at 68.09%. The cumulative evaporated water volume during this period reached 25.81 kg m^−2^, yielding an average evaporation rate of 2.87 kg m^−2^ h^−1^ (Figure 5C). The decrease in evaporation rate can be attributed to two factors: the fluctuating intensity of sunlight (0.91 kW m^−2^, less than 1 sun) and vapor condensate obstructing a portion of the sunlight. The primary objective of the evaporator is to produce sufficient fresh water that meets drinking standards. Analysis of Na^+^, Mg^2+^, K^+^, and Ca^2+^ ion concentrations in simulated seawater and condensate showed that condensate results were well below WHO drinking water ion concentration standards^[^ ^6b^ ^]^ (Figure 5D). Moreover, the total concentration in the condensate was significantly lower than the WHO ion concentration standards (Figure 5E). Subsequently, evaporation tests spanning 9 h per day for 7 days were conducted using simulated seawater and 20 wt.% seawater (Figures S25–S27, Supporting Information). The daily water collection of FJE is primarily correlated with local sunlight intensity and environmental temperature, culminating in a total water collection of 141.75 kg m^−2^ over the 7‐day outdoor evaporation period (Figure 5F), with an average daily collection of 23.24 kg m^−2^. These findings indicate the exceptional stability and salt‐resistant nature of FJE under actual evaporation conditions, even with extremely high salt concentrations.

### 2.6 Salt Resistance and Thermal Localization Mechanism of FJE

**Figure 6:**
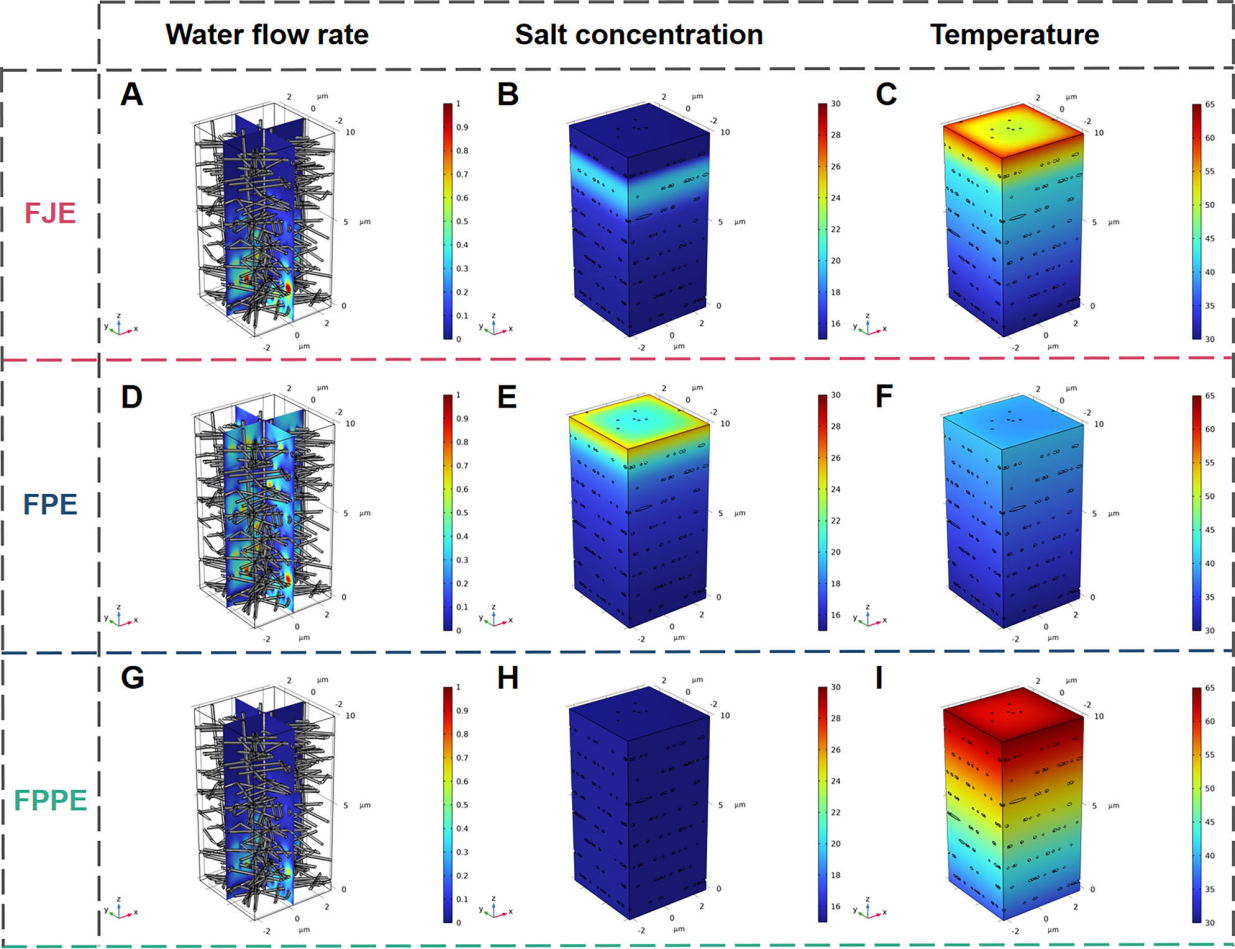
Modeling of water flow, salt concentration, and temperature in three types of evaporators. Simulation of FJE A–C), FPE D–F) and FPPE G–I) in terms of water flow rate, salt concentration distribution, and temperature distribution in an aqueous environment.

To elucidate the mechanisms driving the enhancement of thermal insulation and salt resistance in FJE, three distinct structural configurations were meticulously 3D modeled using Finite Element Analysis. These models were tailored to synchronize the transverse layers with the Janus architecture. Subsequent simulations encompassed water flow dynamics, water pressure variations, salt concentration profiles, and temperature distributions^[^ ^1^, ^7^, ^27^ ^]^ (Figure S28, Supporting Information). The first configuration entailed transverse layers combined with a Janus structure (FJE, **Figure** **6**A–C). The second configuration involved transverse layers coupled with a fully hydrophilic structure (FPE, Figure 6D–F), while the third configuration featured transverse layers integrated with an entirely hydrophobic structure (FPPE, Figure 6G–I). Initially, the water flow rates of FJE and FPE were observed to be partially maintained within the pore channels at 1 µm s^−1^ (Figure 6A,D). This phenomenon stemmed from the fiber's superhydrophilic nature and capillary action, resulting in a substantial increase in flow rate.

Conversely, other regions exhibited a reduced flow rate of 0.2 µm s^−1^, attributed to the looser structure between the evaporator layers. Notably, the thickness of the evaporator influenced water pressure dynamics, displaying a gradient decrease. FPE exhibited a high negative water pressure gradient, while FJE demonstrated a lower negative water pressure gradient (Figure S29, Supporting Information), favoring efficient water exchange.^[^ ^7a^ ^]^ Under a standardized environmental salt concentration of 15 wt.%, FJE exhibited a relatively elevated salt concentration at the hydrophilic‐hydrophobic interface, reaching ≈20 wt.% (Figure 6B). However, due to its high water flow rate and low negative water pressure, FJE facilitated rapid exchange of high‐concentration brine with the lower‐concentration brine beneath, thereby mitigating interfacial salt crystallization through dilution. Conversely, the salt concentration of FPE increased with distance upward, with the top layer registering a salt concentration of 26 wt.%, surpassing the crystallization threshold and predisposed to crystallization^[^ ^6^, ^19^ ^]^ (Figure 6E). This was attributed to the top layer of FPE being too distant from the bottom water, leading to a lack of water exchange.^[^ ^28^ ^]^ Temperature analysis revealed that FJE, featuring transverse layers with a Janus structure, exhibited a temperature decrease from 65 to about 50 °C in the top hydrophobic layer and only 30–35 °C in the hydrophilic layer (Figure 6C). This indicated a significant heat blockage in the hydrophobic layer, enhancing energy utilization efficiency.^[^ ^22^ ^]^ In contrast, FPE, lacking the Janus structure, absorbed a considerable amount of heat due to heating and evaporation across its entirety, resulting in its temperature decreases (Figure 6F), continuous salt concentration increases, and potential crystallization at the top surface (Figure 6E).

Finally, the 3D model simulations of entirely hydrophobic FPPE confirmed the thermal insulation of the transverse hierarchical structure.^[^ ^29^ ^]^ Despite its zero water flow rate, water pressure, and salt concentration (Figure 6G,H; Figure S28, Supporting Information), the temperature distribution simulation highlighted the excellent thermal insulation effect of the transverse layer (Figure 6I). FPPE, devoid of a hydrophilic layer, minimized heat loss from water exchange, achieving a maximum surface temperature of 65 °C and a bottom temperature of only 35 °C. The congruence between simulated temperatures and actual measured temperatures (Figure 3D), along with reasonable water flow, water pressure, and salt concentration distributions, verifies the mechanism of thermal localization and salt resistance achieved by the combination of transverse layers and Janus structure in FJE.

## 3 Conclusion

This study successfully achieved the swift and uncomplicated preparation of large‐area FJE utilizing readily available natural FSC, coupled with soaking and spraying techniques. The inherent transverse hierarchical and Janus structures of FJE allow it to exhibit outstanding capability in continuous salt crystallization resistance and thermal localization within the evaporator, leading to remarkable seawater evaporation and rendering it highly practical for real‐world collection of water from seawater. Moreover, its exceptional cost‐effectiveness and robust mechano‐chemical stability make it possible for its widespread deployment. Simply built from modified natural FSC with scalable production, FJE represents an ideal solar seawater evaporator that opens a novel avenue to scalable manufacturing and pragmatic utilization of sustainable solar evaporation technologies.

## Conflict of Interest

The authors declare no conflict of interest.

## Author Contributions

T.W. and Q.C. contributed equally to this work. Conceptualization was done by T.W., Q.C., C.M., and M.Y. Methodology was developed by T.W., Q.C., L.L., Q.W., Q.W., R.S., Z.X., J.W., and Y.S. Investigation was carried out by T.W. and Q.C. The original draft was written by T.W., Q.C., and L.L.; review and editing were performed by T.W., Q.C., C.M., and M.Y. Funding acquisition was managed by Q.W., Z.X., J.W., Y.S., and M.Y. Resources were provided by C.M. and M.Y. Supervision was handled by C.M. and M.Y.

## Supporting information



Supporting Information

Supplemental Video 1

Supplemental Video 2

Supplemental Video 3

## Data Availability

The data that support the findings of this study are available from the corresponding author upon reasonable request.
