# Supplementary material for: Large‐Scale Flat Silk Cocoons as a Highly Effective Salt‐Resistant Low‐Cost Solar‐Powered Evaporator
Source: Adv Sci (Weinh). 2025 Sep 15;12(41):e11284. doi: 10.1002/advs.202511284 (PMC12591148; doi:10.1002/advs.202511284)
Supplement: Supplementary file 1 — Supporting Information [file ADVS-12-e11284-s001.pdf]

Supporting Information

Large-Scale Flat Silk Cocoons as a Highly Effective Salt-Resistant Low-Cost Solar-Powered Evaporator

Tiancheng Wei†, Qichao Cheng†, Leihao Lu, Quan Wan, Qi Wu, Ruixi Shao, Zongpu Xu, Jie Wang, Yajun Shuai, Chuanbin Mao, Mingying Yang**

† These authors contributed equally to this work.

Tiancheng Wei, Qichao Cheng, Leihao Lu, Quan Wan, Qi Wu, Ruixi Shao, Zongpu Xu, Jie Wang, Yajun Shuai, Mingying Yang

Key Laboratory of Silkworm and Bee Resource Utilization and Innovation of Zhejiang Province, Institute of Applied Bioresource Research, College of Animal Science, Zhejiang University, Hangzhou, Zhejiang, 310058, P. R. China
E-mail: yangm@zju.edu.cn

Chuanbin Mao
Department of Biomedical Engineering, The Chinese University of Hong Kong, Sha Tin, P. R. China
E-mail: cmao@cuhk.edu.hk

Materials and Methods

1. Materials

Flat silk cocoons were obtained from the Department of Animal Science, Zhejiang University. Pyrrole (py), n-hexane, and 4-methylbenzene-sulfonic acid sodium salt (96%) were supplied by Aladdin Industrial Corporation, Shanghai, China. Sinopharm Chemical Reagent Co., Ltd., Shanghai, China supplied NaCl, $FeCl_3$, Phosphoric acid, Rhodamine B, Methylene blue, and Ethanol. Polydimethylsiloxane (PDMS, Sylgard 184) was supplied by Dow Corning Corporation (Shanghai, China). Flat silk cocoons were cut into 50 mm×50 mm uniform blocks, washed with ethanol and deionised water several times, then placed in an oven and dried at 50°C for further processing.

2. Large-scale fabrication of superhydrophilic FSCPn

Flat silk cocoons are formed by using a mass of 5th-instar larvae of (*Bombyx mori*) silkworms spinning silk continuously on a flat surface, and large-scale fabrication of flat cocoons of controllable dimensions is carried out by controlling the area and duration of spinning (Video S1, Fig. S3).

A flat silk cocoon was placed into a beaker, and 30 mL of deionized water was added, after which py was added to the beaker. The mass of py added varied from group to group; there were 6 groups, and the amount of py added to each group was 0.1, 1, 3, 5, 7, and 10 mmol. After that, 1 mL of 1% phosphoric acid and 1 mmol of 4-methylbenzene-sulfonic acid sodium (PTS) were added, and a sealing shock was carried out for 10 min. Then, the $FeCl_3$ aqueous solution was added according to the molar ratio py: $FeCl_3$=1:1, and finally, the total volume was controlled to 50 mL. After continuous shaking for 2 h, it was removed by standing. After performing ethanol and moisture backwash cleaning, FSCP*n* (*n*=1-10, each representing a specific amount of py (*n* mmol, *n*=1-10) added during fabrication) with different polypyrrole contents was obtained by drying at 50°C.

3. Fabrication of superhydrophobic FSCP*n*-P*m*

n-hexane was premixed with 0.01wt% PDMS (mass of prepolymer: curing agent = 10: 1), and the mixture was treated by ultrasound for 10 min. The PDMS solution was poured into the spray gun, and the FSCP*n* was sprayed with the front side facing upwards at 5 cm. 2 mL of the PDMS solution was sprayed uniformly each time for a total of 1-3 times, leading to FSC with different thicknesses of the hydrophobic layer. The FSCP*n*-P*m* (*m*=1, 2, 3, representing different numbers of PDMS spraying cycles) with different thicknesses of hydrophobic layers were obtained by curing the evaporator in an oven at 80°C for 30 min.

The specific naming rules of evaporators are as follows. For example, FSCP7-P1 signifies an evaporator fabricated with 7 mmol of py and one PDMS spraying cycle. Besides, the FSCP7, FSCP7-P1, and FSCP7-P3 evaporators are termed FPE, FJE, and FPPE, respectively.

4. Characterization

Photographs and videos were taken with a SONY camera (FDR-AX60). A Scanning Electron Microscope (SEM, Zeiss G300) was used to characterise the surface structure and morphology. An energy dispersive spectrometer (EDS) was attached to the SEM to detect the surface composition of samples. SHIMADZU-8400S spectrometer was used to record Fourier transform infrared (FTIR) spectroscopy, and FTIR spectroscopy was used to observe the chemical structure of the specimens. A contact angle analyser (Data Physics OCA15, Germany) was used to measure the static contact angle (CA) of the water. The volume of water droplets used to measure the static contact angle was 8 μl. Thermal imaging was taken using a thermal imaging camera (FLIR-E6390). Optical absorbance is measured by a UV-VIS-NIR spectrophotometer (Shimadzu UV3600). Air Permeability Testing was carried out using a Digital Air Permeability Tester (YG461E). Mechanical strength is tested by a universal testing

machine (AGS-J). The simulated sunlight for the light tests was obtained from a xenon lamp (CME-Xe300F). Light intensity was detected by a solar power meter (SM206-SOLAR). Longitudinal thermal conductivity was measured using a laser thermal conductivity meter (NETZSCH LFA 457 Micro Flash). DSC analysis was measured using a differential scanning calorimeter (TGA2, METTLER TOLEDO). Porosity and Specific Surface Area were measured by Mercury Piezometer (Micromeritics 9605). Ion concentration was detected by inductively coupled plasma mass spectrometer (NexION 2000).

5. Indoor and outdoor light evaporation tests

Indoors, the evaporator was placed on 500 mL of brine, separated by polystyrene foam, and connected to the water flow through filter paper. A xenon lamp was used to provide simulated sunlight of various light intensities. Other liquids (0.1 M HCl, 0.1 M NaOH) were tested similarly.

Outdoor: Highly translucent customized glass containers were used with water and evaporators in intermediate tubes. The condensate was collected and weighed through a ramp or hood.

6. Experiments and calculations of evaporation efficiency

The evaporation efficiency η can be calculated by Equation 1[1]:

$$\eta = \frac{\dot{m}\, E_{equ}}{C_{opt}\, P_0}$$

1

$\dot{m}$ is the evaporation rate, E_{equ} is the equivalent evaporation enthalpy of the aqueous phase change of the evaporator in water, C_{opt} is the light concentration value, and P_0 is one solar power intensity (1 kW m^{-2}). To obtain the E_{equ} of the evaporator, a water evaporation test was carried out through a dark environment to ensure the same energy input (U_{in}). This was done by placing pure water and an evaporator of the same evaporation area in separate airtight containers containing the exact weight of saturated potassium dihydrogen phosphate solution. Then, 1-hour dark environment evaporation was carried out, and the mass change was detected; the evaporation temperature and pressure were 26°C and one atmosphere pressure[2], respectively.

Equivalent evaporation enthalpy (E_{equ}) can be calculated using Equation 2[3]:

$$U_{in} = E_0 m_0 = E_{equ} m_g \quad 2$$

E_0 is the equivalent evaporation enthalpy of pure water, the E_0 should be 2439.50 kJ kg^{-1} at 26.0 °C (for pure water)[4]. m_0 is the mass change of the pure water group, and m_g is the mass change in the evaporator group, E_{equ} is the enthalpy of water's evaporation in the evaporator group. Figure S14 shows the results of mass change, equivalent evaporation enthalpy, and evaporation efficiency for different groups in the dark.

7. Calculation of heat loss

Heat loss from interfacial evaporation is generally divided into three components, including radiation (P_{rad}), convection (P_{conv}) and conduction (P_{cond}). The equations for the three components are as follows:

The heat loss by radiation (Φ) is generally calculated using the Stefan-Boltzmann Equation3[5]:

$$P_{\mathrm{rad}} = A\, \varepsilon\, \sigma\, (T_1^4 - T_2^4) \quad 3$$

where P_{rad} is the radiation heat flux, A is the surface area of the actual evaporator evaporating at the evaporation interface (A =9 m^{-2}), ε is the emissivity of the evaporation interface material (0.98), and σ is the Stephen Boltzmann constant (5.6703 × 10-8 W m^{-2} K^{-4}). T_1 is the surface temperature of the evaporator in one sunlight (T_1 =327.95 K for the FJE), and T_2 is the evaporator surface vapor temperature (T_2 = 325.65 K for FJE).

The radiation heat loss of the FJE was calculated to be 17.85 W m^{-2}. The radiation heat losses of the FPE and FPPE were 38.18 W m^{-2} and 196.85 W m^{-2}, respectively.

Heat loss by thermal convection is generally calculated using Newton's Law of Cooling Equation 4[3]:

$$P_{\text{conv}} = A\,h\,(T_1 - T_2) \tag{4}$$

where P_{conv} is the convection heat flux, A is the surface area of the actual evaporator evaporating at the evaporation interface (A =9 m^{-2}), h is the natural convective heat transfer coefficient, determined empirically from previous research (5 W m^{-2} K^{-1}), T_1 is the surface temperature of the evaporator in one sunlight (T_1 =327.95 K for the FJE), and T_2 is the evaporator surface vapor temperature (T_2 = 325.65 K for FJE). The convection heat loss of the FJE was calculated to be 11.5 W m^{-2}. The convection heat losses of the FPE and FPPE were 28.5 W m^{-2} and 125 W m^{-2}, respectively.

The primary heat loss of the interface evaporator was focused on the heat conduction between the evaporator and the bulk water, which was a hot research topic for solving heat loss. The heat loss by thermal conduction was calculated by the Equation 5[6]:

$$P_{\text{cond}} = m\,C\,\Delta T \tag{5}$$

where P_{cond} is the conduction heat flux, m is the total mass of bulk water used in evaporation (500 g), C is the specific heat capacity of water (4.2 J K^{-1} g^{-1}), and ΔT is the temperature change of the bulk water before and after 1 h of continuous evaporation (ΔT=0.2 K for the FJE). The conduction heat loss of the FJE was calculated to be 129.62 W m^{-2}. The convection heat losses of the FPE and FPPE were 259.85 W m^{-2} and 64.8 W m^{-2}, respectively.

The heat loss calculations for FJE, FPE, and FPPE (Fig. S15, Table S1) show that FJE reduces the convection heat loss by 50% compared to the significant conduction heat loss of FPE. Meanwhile, most of the heat loss of FPPE was concentrated in thermal radiation due to their large thickness. The sum of evaporation energy and heat loss was slightly different from the input energy (20 W m^{-2}), which could be attributed to errors in temperature determination and heat loss from contact with the polystyrene foam.

8. Simulation calculation of 3D models

The 3D models of FJE, FPE, and FPPE were constructed using COMSOL Multiphysics software (5.6 Version), and three items were computationally simulated for the three models, including water pressure & water flow, salt concentration distribution, and temperature distribution[2].

By the model already constructed, the evaporator has a capillary action for water absorption. Therefore, calculations of water pressure and water flow could be performed using the following Equations:

$$\rho \boldsymbol{\nabla} \cdot \boldsymbol{u} = \nabla \cdot [-p\boldsymbol{I} + \boldsymbol{K}] + F \tag{6}$$

$$\rho \nabla \cdot \boldsymbol{u} = 0 \tag{7}$$

$$\boldsymbol{K} = \mu(\nabla \boldsymbol{u} + (\nabla \boldsymbol{u})^T) \tag{8}$$

$$0 = \nabla \cdot [-p\boldsymbol{I} + \boldsymbol{K}] + \boldsymbol{F} + \rho \boldsymbol{g} \tag{9}$$

Where ρ represents the mass density, $\boldsymbol{u}$ is the velocity vector, p is pressure, $\boldsymbol{I}$ is the second-order unit tensor, $\boldsymbol{K}$ is the viscous stress tensor, F is the gravity of water, μ is the viscosity of water, T is the temperature of water.

Heat transfer is based on the evaporator, the evaporator, the water, and the water itself so heat transfer can be divided into two parts: 1, solid heat transfer and 2, liquid heat transfer. The two heat transfer modules, solid and liquid, are coupled through the software, where the solid heat transfer can be calculated using the following Equations:

$$\rho Cp\boldsymbol{u} \cdot \nabla T + \nabla \cdot \boldsymbol{q} = Q + Qted \tag{10}$$

$$\boldsymbol{q} = -k\nabla T \tag{11}$$

$$Q_{ted} = -\alpha T{:}\frac{dS}{dT} \qquad 12$$

Where ρ is the density, Cp is the specific heat capacity at constant pressure, $\boldsymbol{u}$ is the velocity vector, T is the absolute temperature, $\boldsymbol{q}$ is the heat flux by conduction, Q contains additional heat sources, Q_{ted} is the thermoelastic damping and accounts for thermoelastic effects in solids, k is the thermal conductivity (a scalar or a tensor when the thermal conductivity is anisotropic), α is the coefficient of thermal expansion, S is the second Piola-Kirchhoff stress tensor.

In addition, the heat transfer of the fluid can be calculated by the following equations:

$$\rho C_p \boldsymbol{u} \cdot \nabla T + \nabla \cdot \boldsymbol{q} = Q + Qp + Qvd \qquad 13$$

$$\boldsymbol{q} = -k \qquad 14$$

$$\rho = \frac{p_A}{R_S T} \qquad 15$$

$$Q_p = \alpha_p T(\frac{\partial p}{\partial t} + \boldsymbol{u} \cdot \nabla p) \qquad 16$$

$$Q_{vd} = \tau{:}\,\nabla \boldsymbol{u} \qquad 17$$

Where ρ is the density, C_p is the specific heat capacity at constant pressure, T is the absolute temperature, k is the thermal conductivity (a scalar or a tensor when the thermal conductivity is anisotropic), $\boldsymbol{q}$ is the heat flux by conduction, Q contains heat sources other than viscous dissipation, Q_p is the pressure changes due to the work and is the result of heating under adiabatic compression as well as some thermoacoustic effects, Q_{vd} is viscous dissipation in the fluid, α_p is the coefficient of thermal expansion, p is the pressure, τ is the viscous stress tensor.

To explain the salt resistance mechanism of the evaporators, we simulated the diffusion of ions between the internal layers of the three evaporators to observe the salt concentration distribution inside the evaporators. We set the salt concentration at 15 wt%. The mass transfer Equations can simulate the salt distribution:

$$\nabla \cdot \boldsymbol{Jj} + \boldsymbol{u} \cdot \nabla cj = Rj \qquad 18$$

$$\boldsymbol{Jj} = -Dj \nabla cj \qquad 19$$

Where $\boldsymbol{Jj}$ is the mass flux relative to the mass averaged velocity, $\boldsymbol{u}$ is the mass-averaged velocity vector, cj is the concentration of the species, Rj is a reaction rate expression for the species, Dj is the diffusion coefficient.

9. Statistical Analysis

All data are presented as mean ± standard deviation, data are analyzed using Origin 2025 and Graphpad Prism 8. The error bar generally represents the standard mean error and experiments are generally performed n = 3.

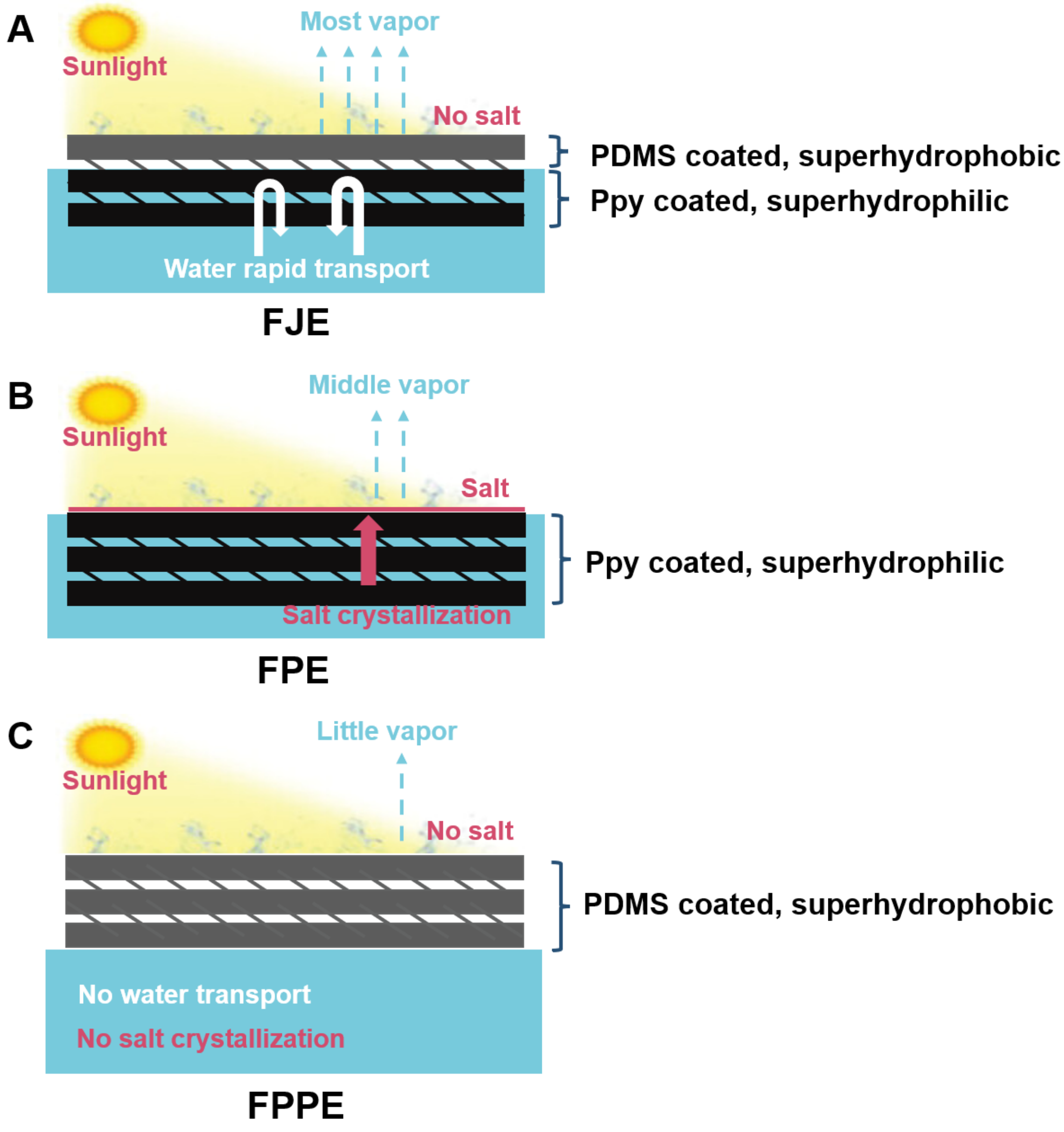

Fig. S1. Differences in composition and evaporation of FJE, FPE, and FPPE.
(A) FJE relies on super-hydrophilic bottom layers for rapid water transport and prevention of salt crystallization as well as on super-hydrophobic top layers for heat shielding and rapid evaporation, so it can produce the most vapor. (B) FPE relies on the overall superhydrophilicity to allow heat dissipation, suffers salt crystallization that affects evaporation, and thus produces a medium level of vapor. (C) FPPE relies on total superhydrophilicity for low heat loss and suffers no risk of crystallization at all, but produces little vapor due to the lack of water contact.

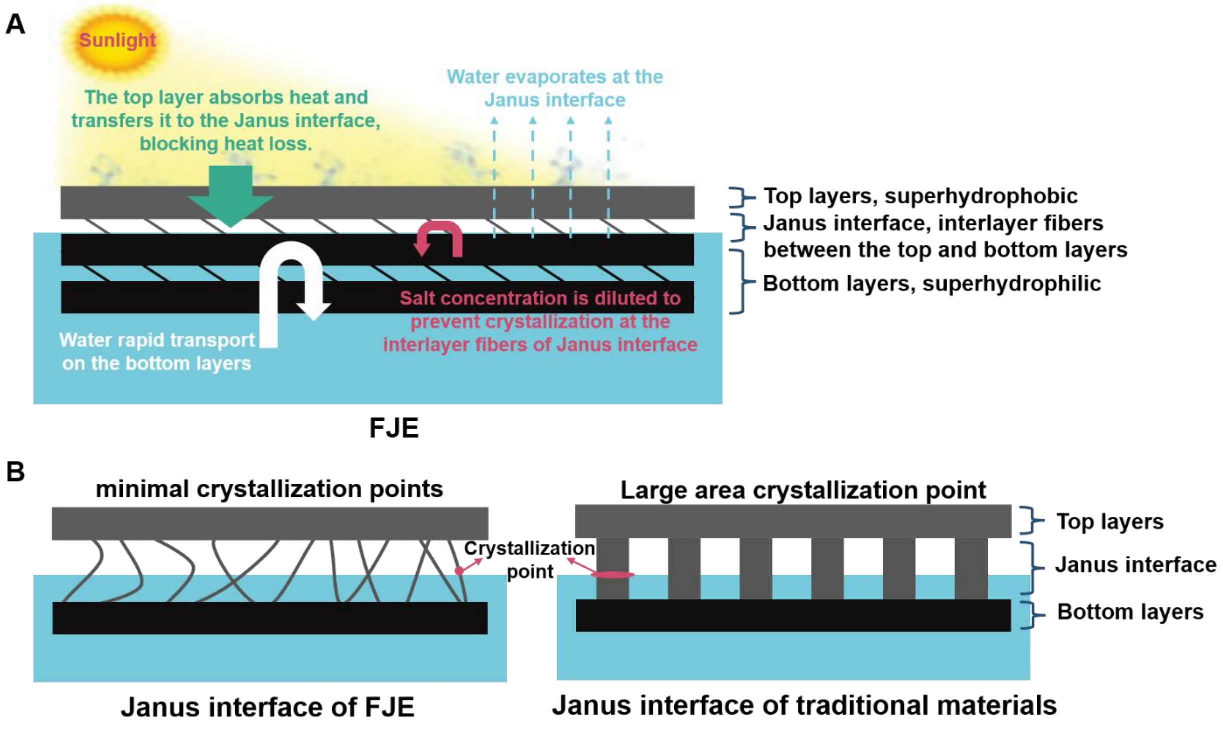

Fig. S2. Mechanism of high evaporation and salt resistance of FJE, difference of Janus interface between FJE and traditional materials

(A) Mechanism of high rate evaporation and salt resistance: The rapid water transportation in the bottom layers reduces the salt concentration and prevents the salt crystallization at the Janus interface. The top layers absorb heat and concentrate heat on water evaporation to reduce heat loss. The air layer and interlayer fibers at the Janus interface reduce the crystallization sites and promote rapid evaporation. (B) The Janus interface of FJE is interlayer fibers with minimal water contact and few salt crystallization sites. The Janus interface of traditional materials is a tubular channel with a large water contact surface and many salt crystallization sites.

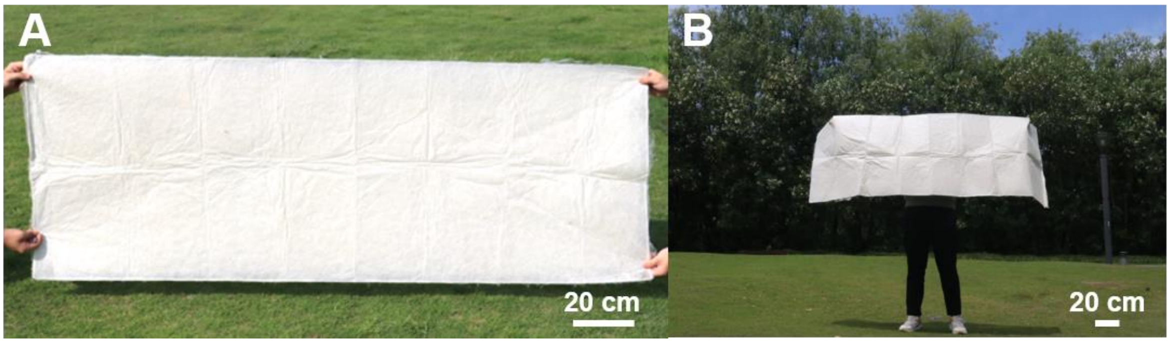

Fig. S3. Photographs of flat silk cocoons prepared on a large area (70*200 cm)

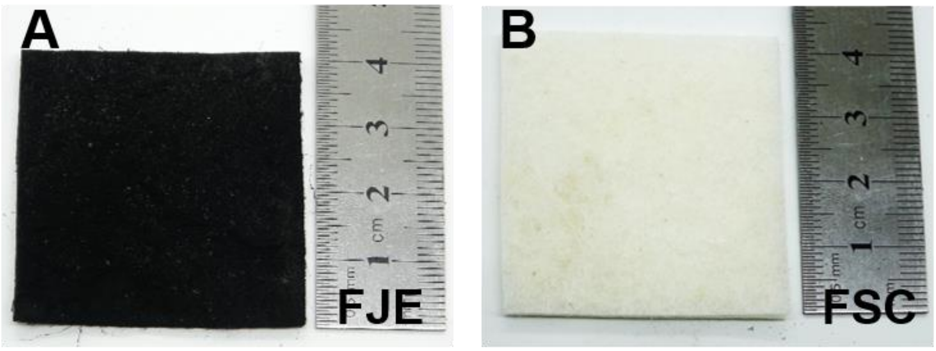

Fig. S4. Optical photographs of (A) FJE (FscP7-P1) evaporator and (B) FSC (flat silk cocoon)

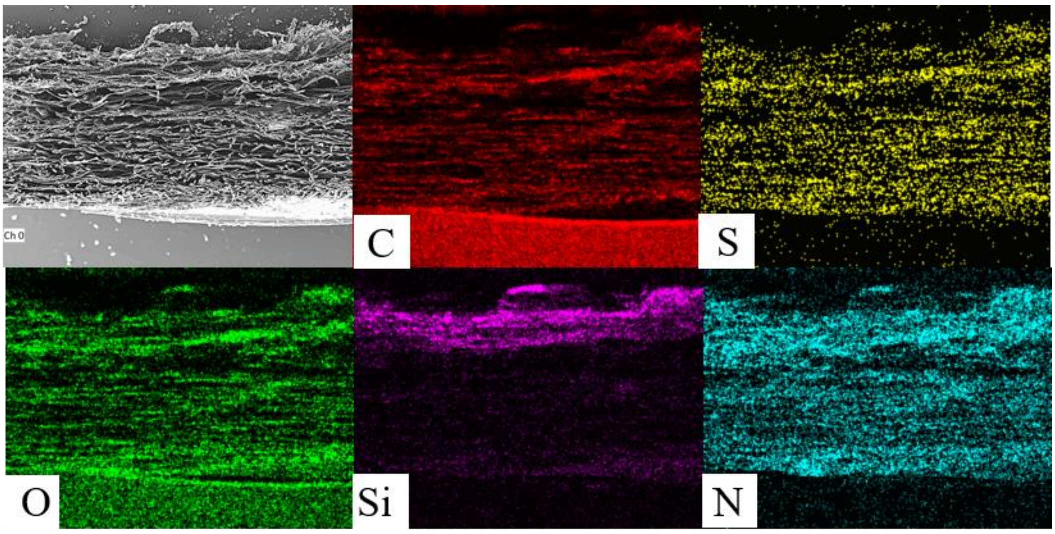

Fig. S5. EDS elemental distribution of FJE (FscP7-P1) solar seawater evaporator

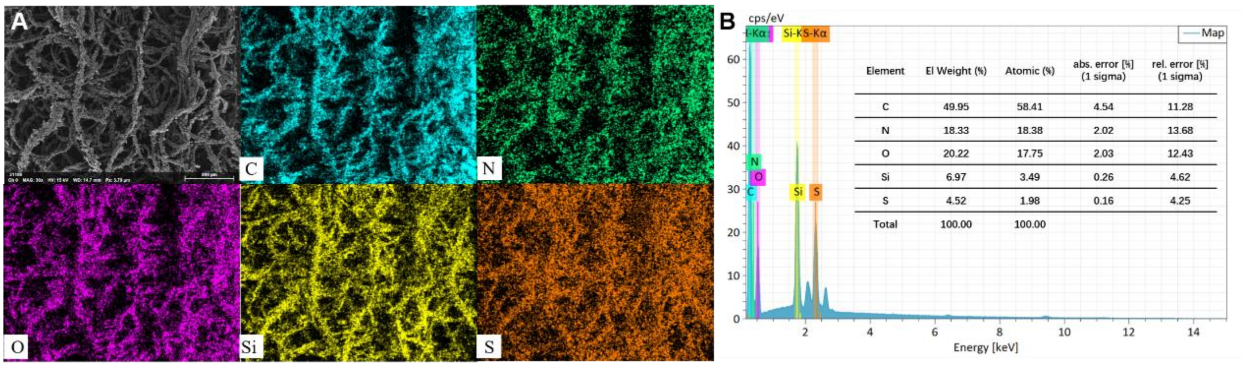

Element	El Weight (%)	Atomic (%)	abs. error [%] (1 sigma)	rel. error [%] (1 sigma)
C	49.95	58.41	4.54	11.28
N	18.33	18.38	2.02	13.68
O	20.22	17.75	2.03	12.43
Si	6.97	3.49	0.26	4.62
S	4.52	1.98	0.16	4.25
Total	100.00	100.00		

Fig. S6. EDS for the top layer of FJE (FscP7-P1).
(A) elemental distribution and (B) detailed information.

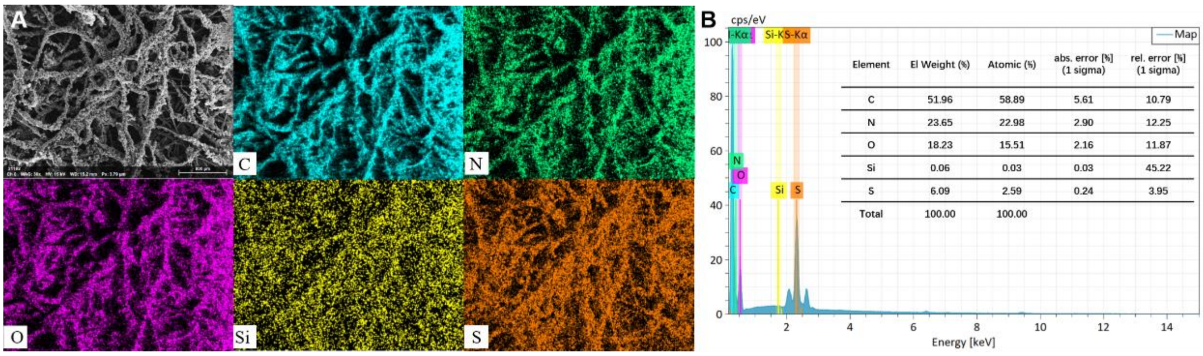

Element	El Weight (%)	Atomic (%)	abs. error [%] (1 sigma)	rel. error [%] (1 sigma)
C	51.96	58.89	5.61	10.79
N	23.65	22.98	2.90	12.25
O	18.23	15.51	2.16	11.87
Si	0.06	0.03	0.03	45.22
S	6.09	2.59	0.24	3.95
Total	100.00	100.00		

Fig. S7. EDS for the bottom layer of FJE (FscP7-P1).
(A) elemental distribution and (B) detailed information.

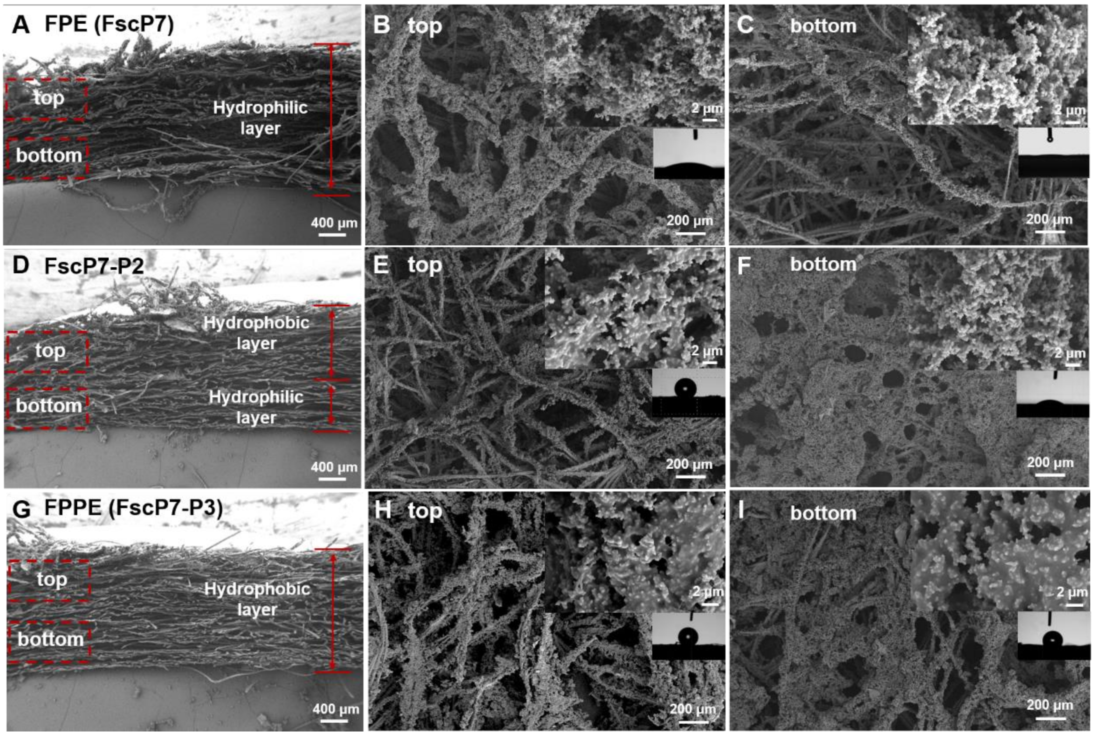

Fig. S8. SEM image of (A-C) FPE (FscP7), (D-F) FscP7-P2, (G-I) FPPE (FscP7-P3). (A, D, G) cross-section, (B, E, H) top view, (C, F, I) bottom view. The inset shows an enlarged SEM image with contact angle photographs.

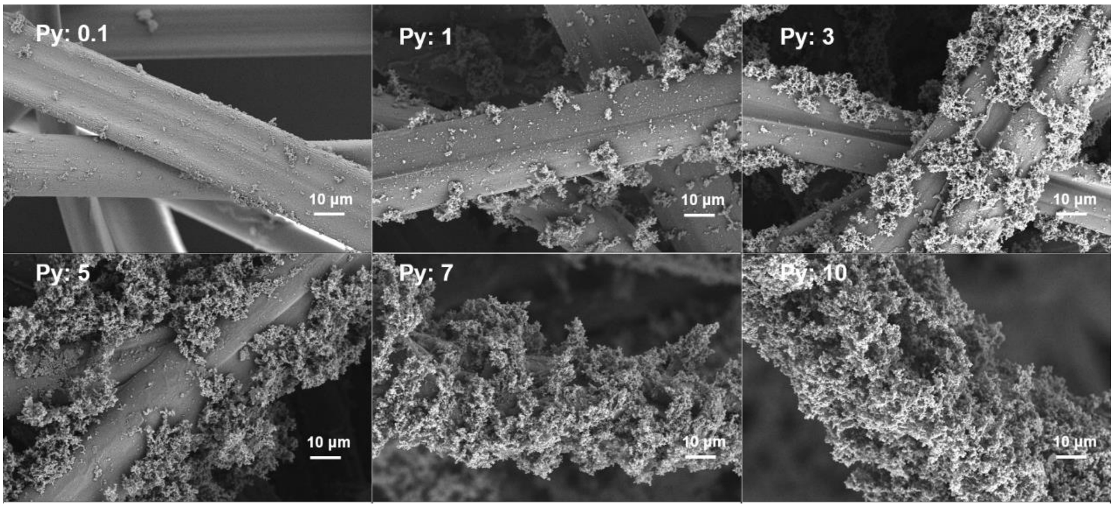

Fig. S9. SEM image of FscP (mass of Py from 0.1 to 10) fibres.

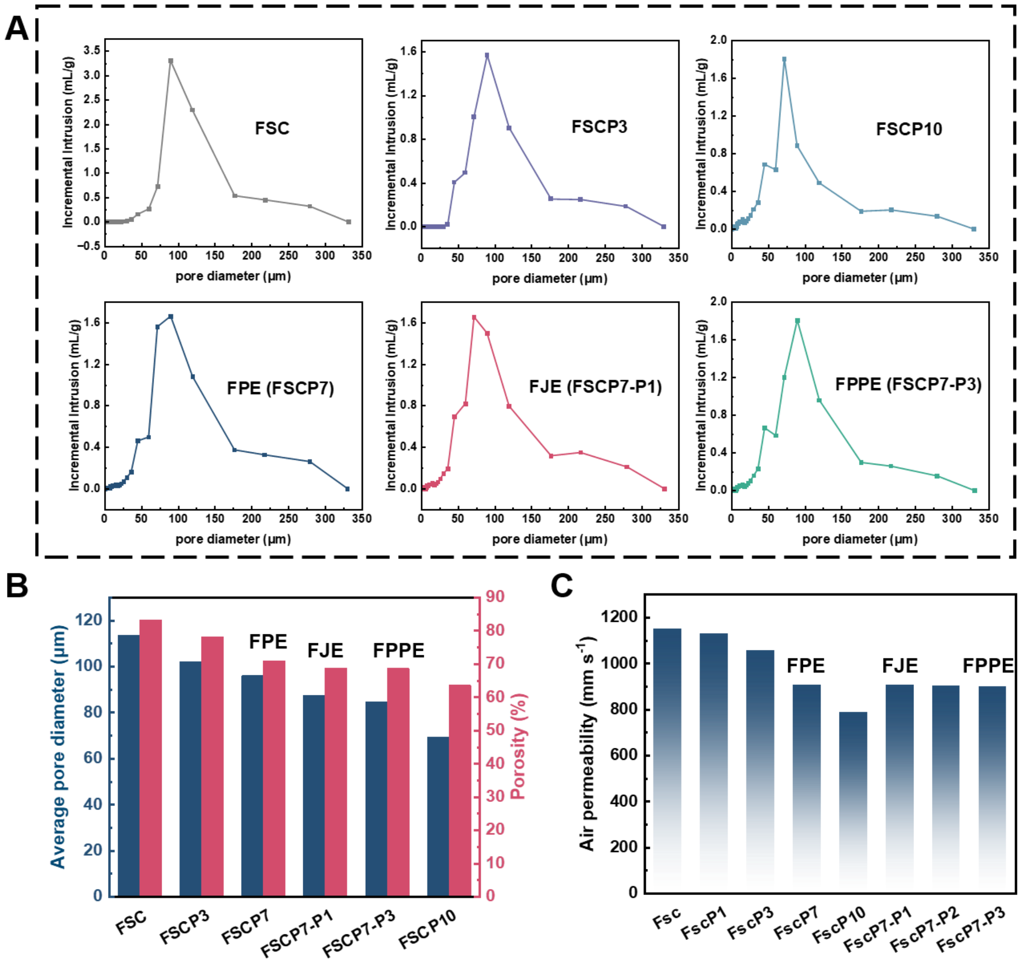

Fig. S10. Air permeability and pore variation of FSC with different PPy and PDMS specific gravity.

(A) pore distribution measured by piezomercury method, (B) calculated mean pore diameter and porosity, (C) air permeability

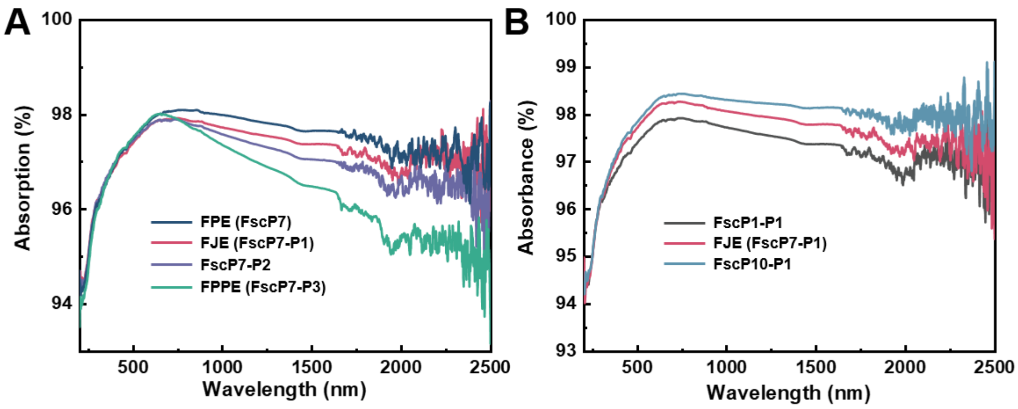

Fig. S11. Detailed comparative data on solar absorption for different evaporator groups. (A) Comparison of evaporator groups with different PDMS coating times, (B) Comparison of evaporator groups with different Ppy content.

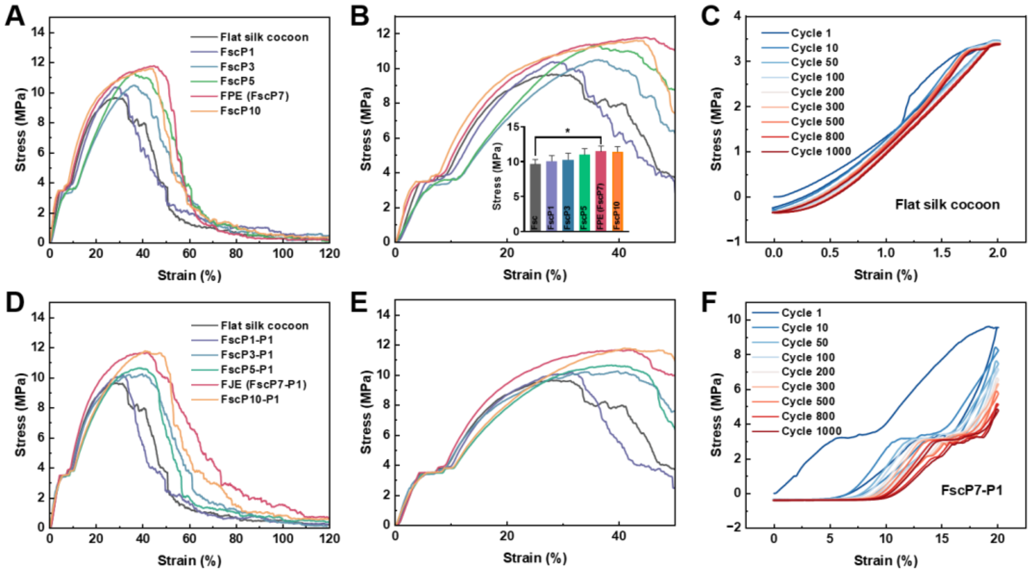

Fig. S12. Mechanical strength of solar seawater evaporation with different component contents.

(A-B) Tensile mechanical strength of flat silk cocoon with different proportions of Ppy content, the insets show the comparison of the maximum tensile strength of different groups. (C) Cyclic tensile strength of flat silk cocoon. (D-E) Tensile mechanical strength of flat silk cocoon with different percentages of Ppy content after PDMS coating. (F) Cyclic tensile strength of FJE (FscP7-P1) with 20% tensile ratio.

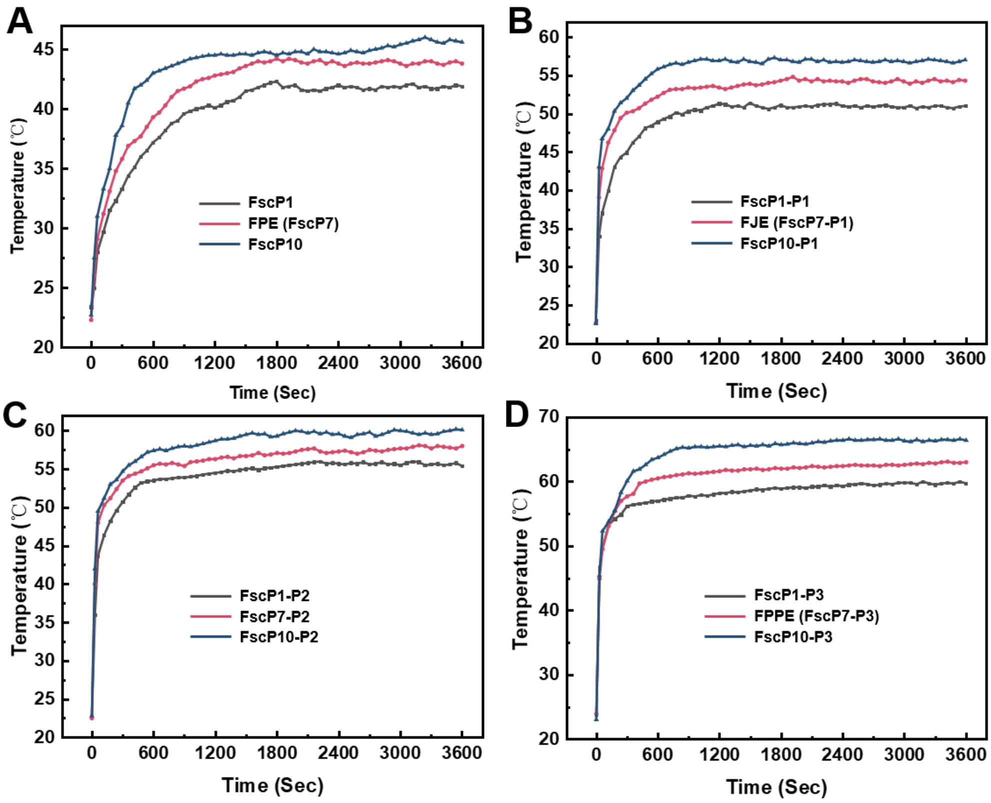

Fig. S13. Temperature of evaporators within the water at one solar intensity for solar seawater evaporators with different component ratios

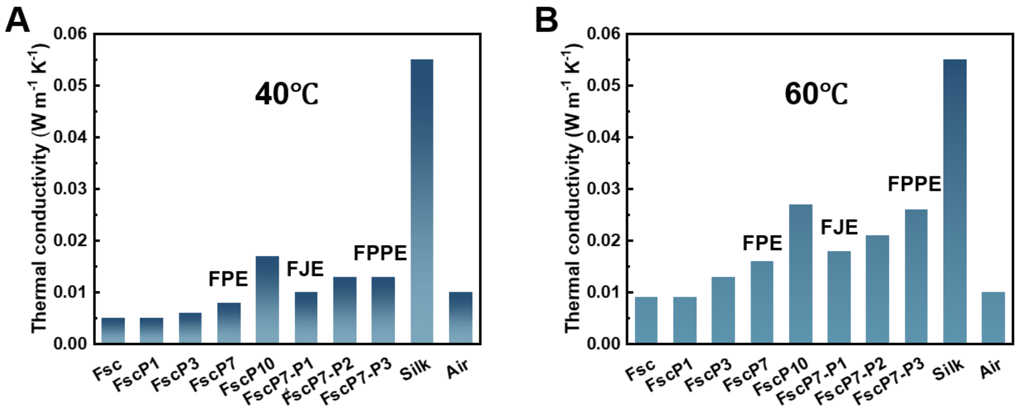

Fig. S14. Thermal conductivity of FSC (flat silk cocoon) with different proportions of PPy and PDMS content in (A) 40℃ and (B) 60℃.

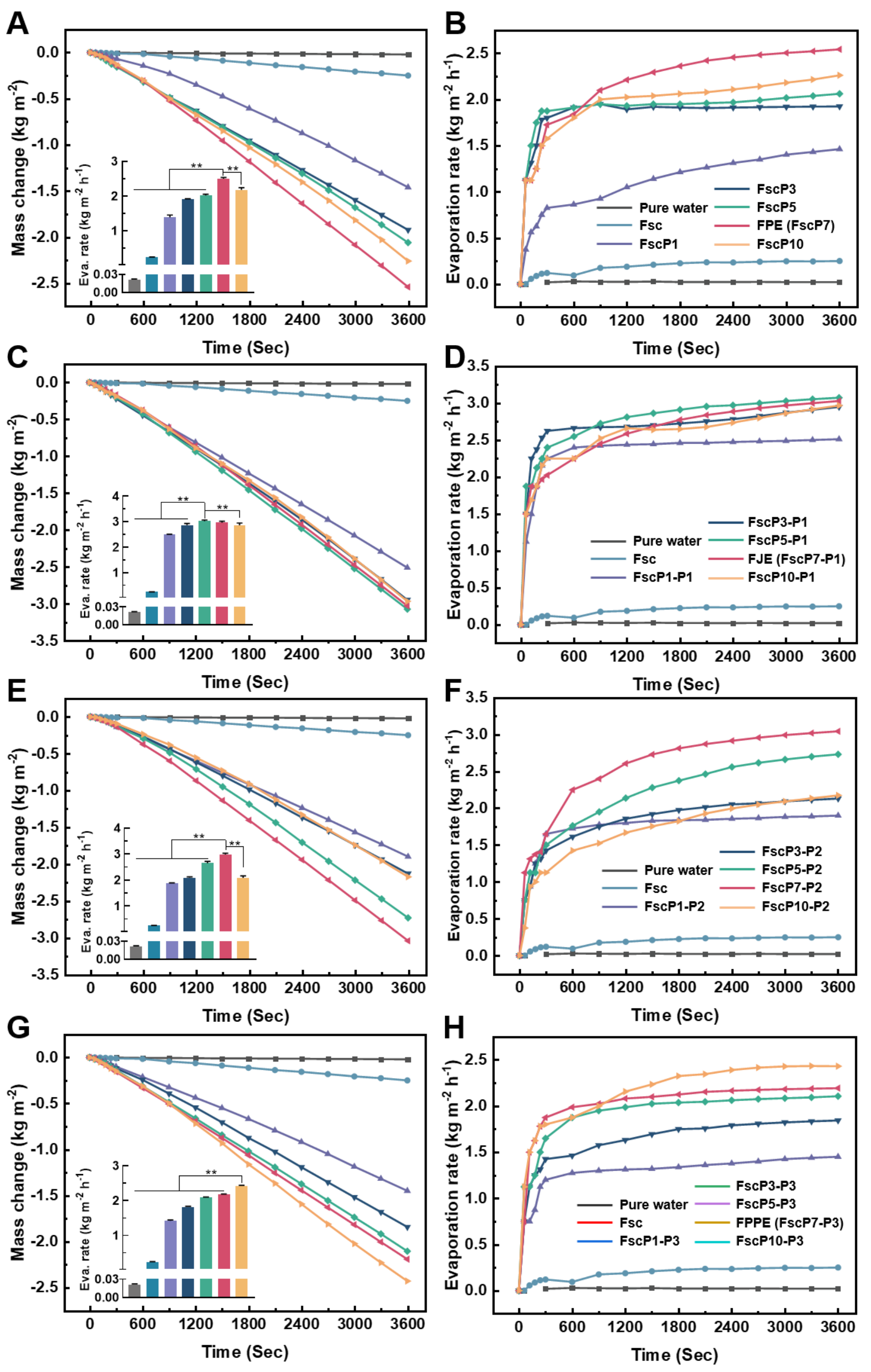

Fig. S15. Summary of evaporation from different evaporators.

Evaporated water (A, C, E, G) mass change and (B, D, F, H) evaporation rate results for solar evaporators with different Ppy and PDMS contents. The inset shows the results of comparing the maximum evaporation rate for each group.

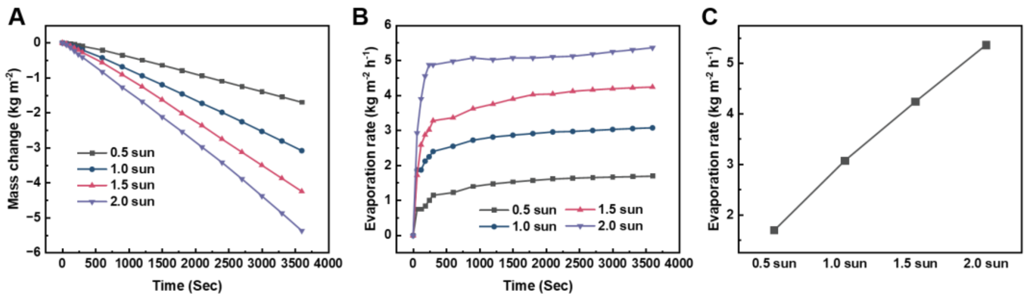

Fig. S16. Effect of different multiples of optical intensity on FJE.

(A) Mass change in FJE (FscP7-P1) evaporation at different multiples of optical intensity, (B) evaporation rate, (C) relationship between optical intensity and evaporation rate

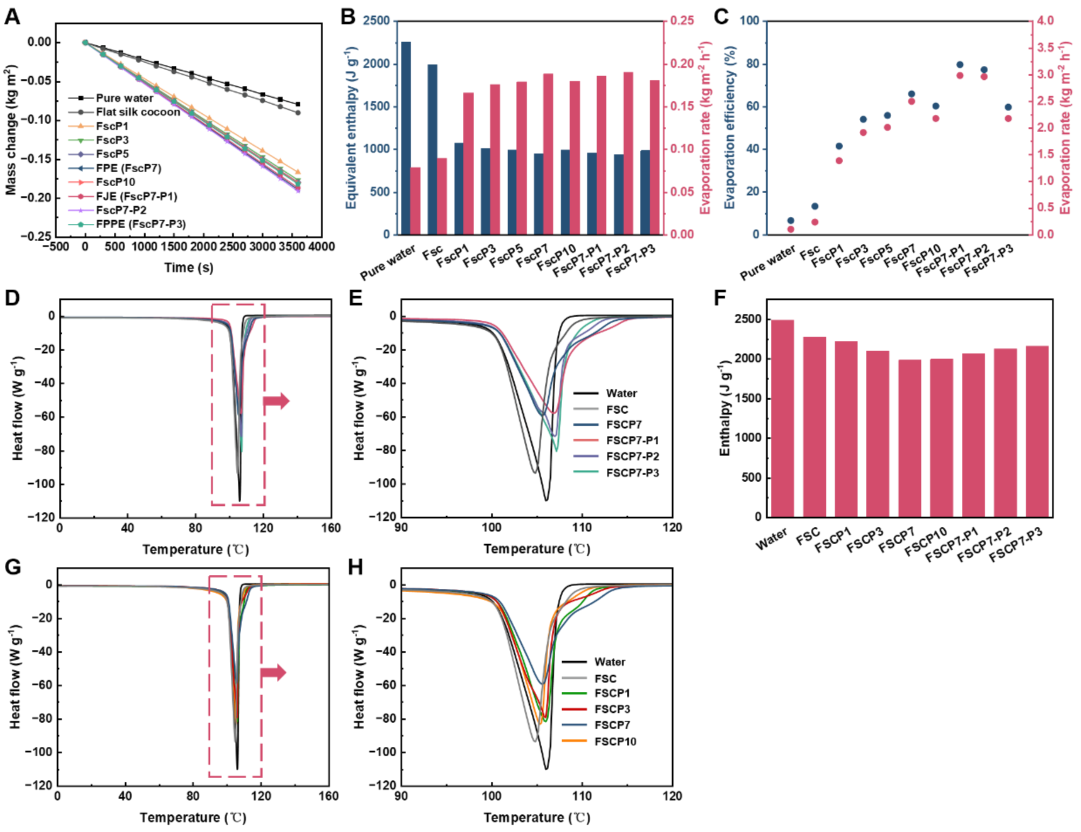

Fig. S17. Calculation of evaporation efficiency of evaporators with different component contents.

(A) mass change of evaporators under dark environment, (B) equivalent enthalpy of water evaporation and evaporation rate under dark environment, (C) evaporation efficiency and evaporation rate, (D, E, G, H) results of DSC test for evaporators with different component contents, (F) statistical results of the high-temperature (0-160°C) water evaporation enthalpy of DSC test.

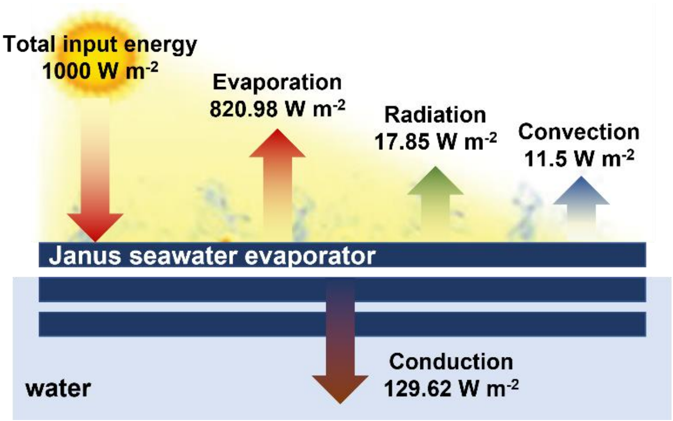

Fig. S18. Energy balance and heat transfer diagram of FJE (FscP7-P1) solar seawater evaporator

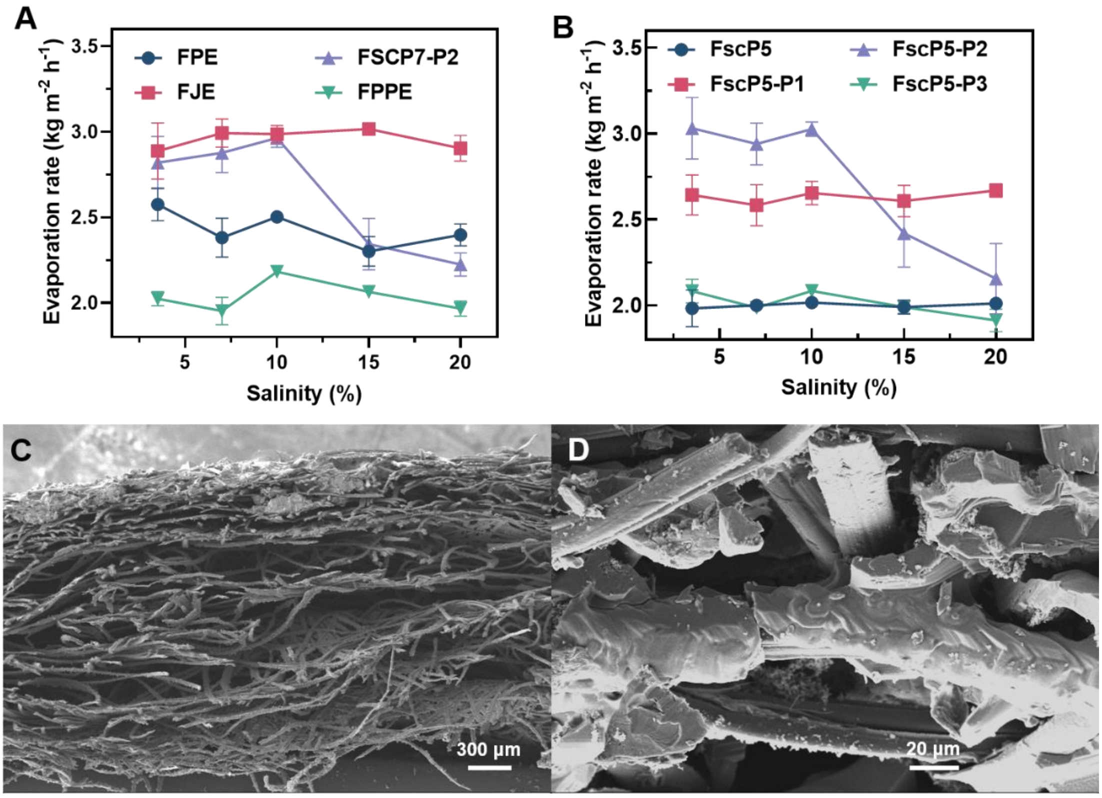

Fig. S19. Crystallization of salt by continuous evaporation of different evaporators and salinity.

Evaporation rate of (A) FscP7 and (B) FscP5 evaporator with different PDMS coatings at different salt concentrations. (C) Cross-sectional part and (D) bottom part of the SEM of FJE (FscP7-P1) after evaporation at 20 wt% salt concentration for 18 h.

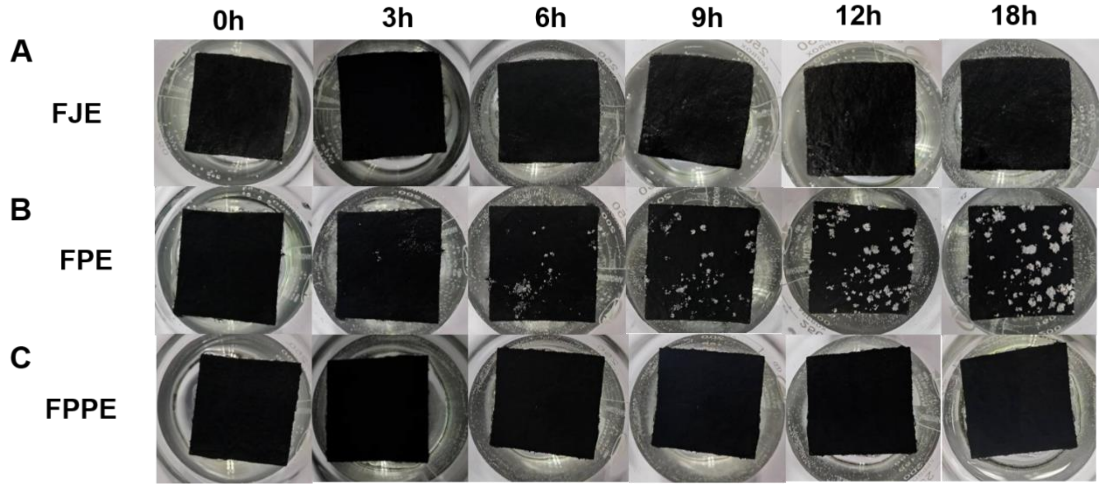

Fig. S20. Photographs of (A) FJE (FscP7-P1), (B) FPE (FscP7), (C) FPPE (FscP7-P3) seawater evaporators for 0-18 hours evaporation at 20 wt% salt concentration.

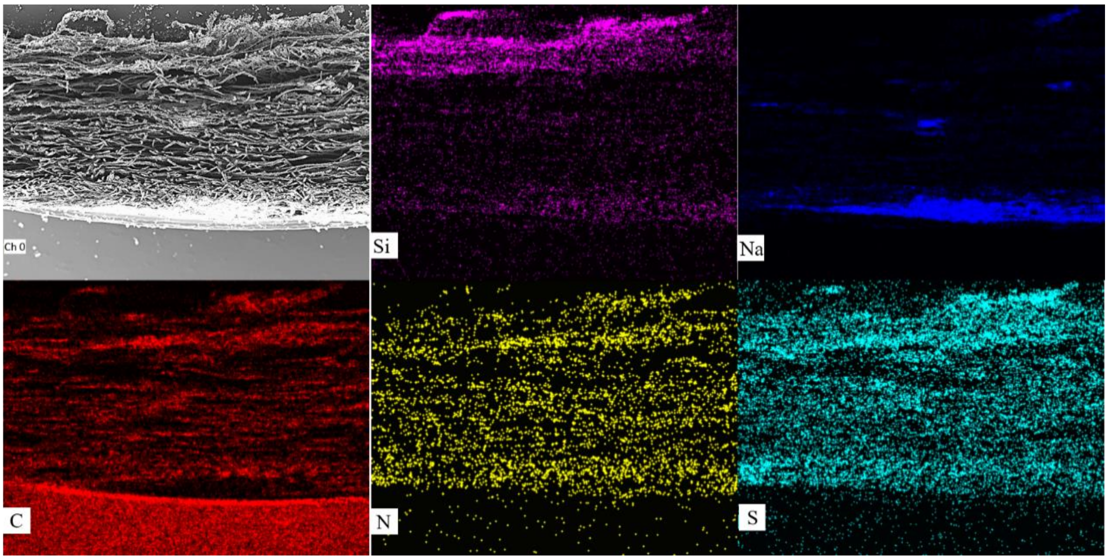

Fig. S21. EDS elemental distribution of FJE (FscP7-P1) after evaporation at 20 wt% salt concentration for 18h.

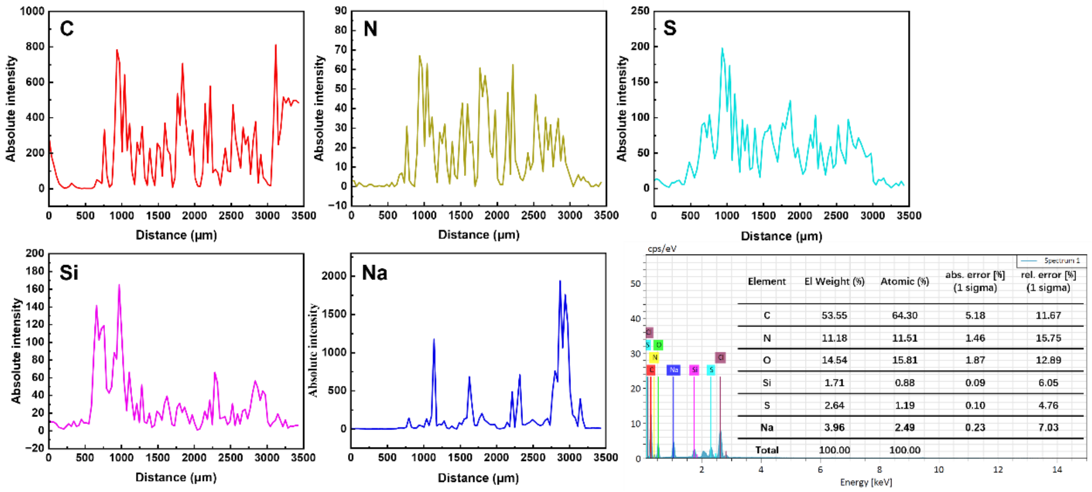

Element	El Weight (%)	Atomic (%)	abs. error [%] (1 sigma)	rel. error [%] (1 sigma)
C	53.55	64.30	5.18	11.67
N	11.18	11.51	1.46	15.75
O	14.54	15.81	1.87	12.89
Si	1.71	0.88	0.09	6.05
S	2.64	1.19	0.10	4.76
Na	3.96	2.49	0.23	7.03
Total	100.00	100.00		

Fig. S22. EDS cross-section scanning elemental distribution of FJE (Fsc7-P1) after evaporation at 20 wt% salt concentration for 18 h. The table shows detailed EDS elements data.

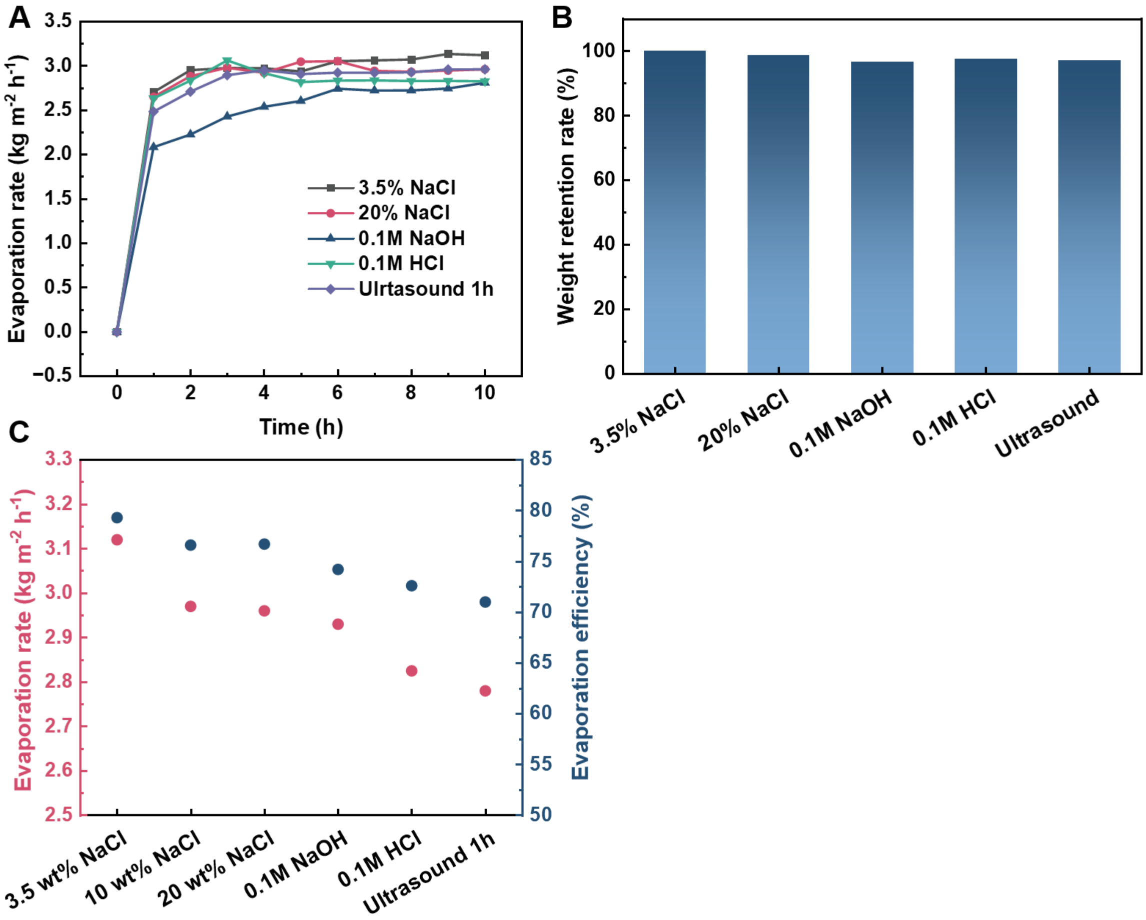

Fig. S23. Stable evaporation of FJE in different environments. (A) Sustained evaporation rate, (B) weight retention rate and (C) evaporation efficiency of FJE (FscP7-P1) in different extreme environments.

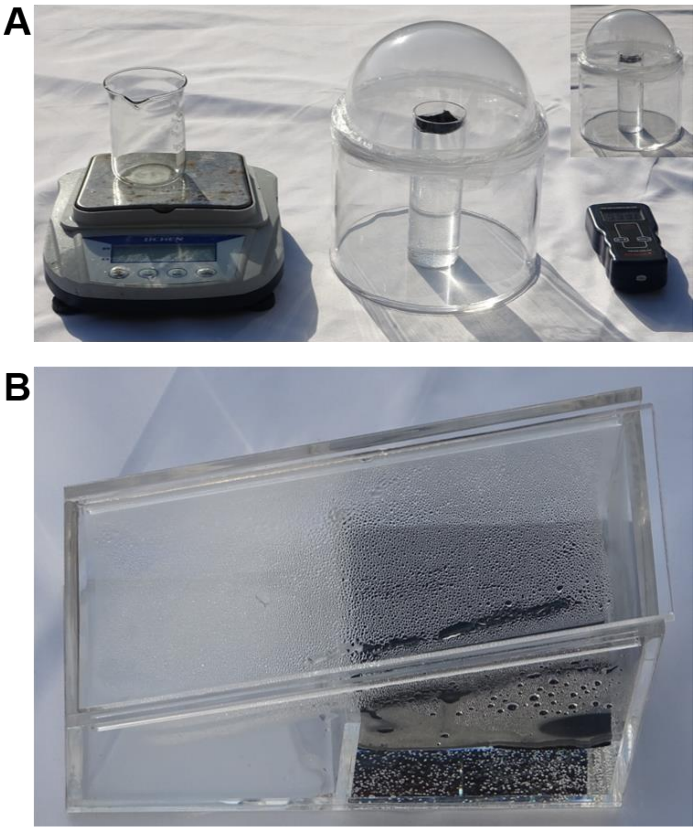

Fig. S24. Photographs of (A) outdoor evaporation test, (B) vapor generation and condensation of continuous evaporation.

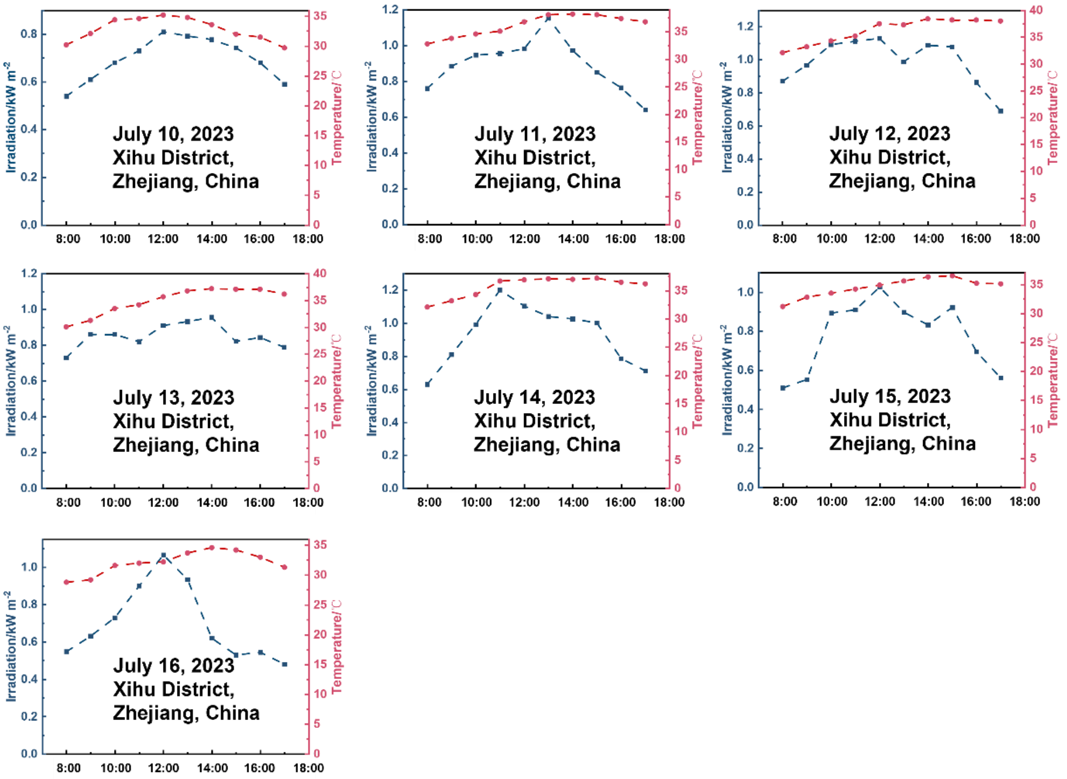

Fig. S25. Details of Temperature and irradiation for 7-day Outdoor Cycling Evaporation Test of FJE (FscP7-P1) Solar Seawater Evaporator.

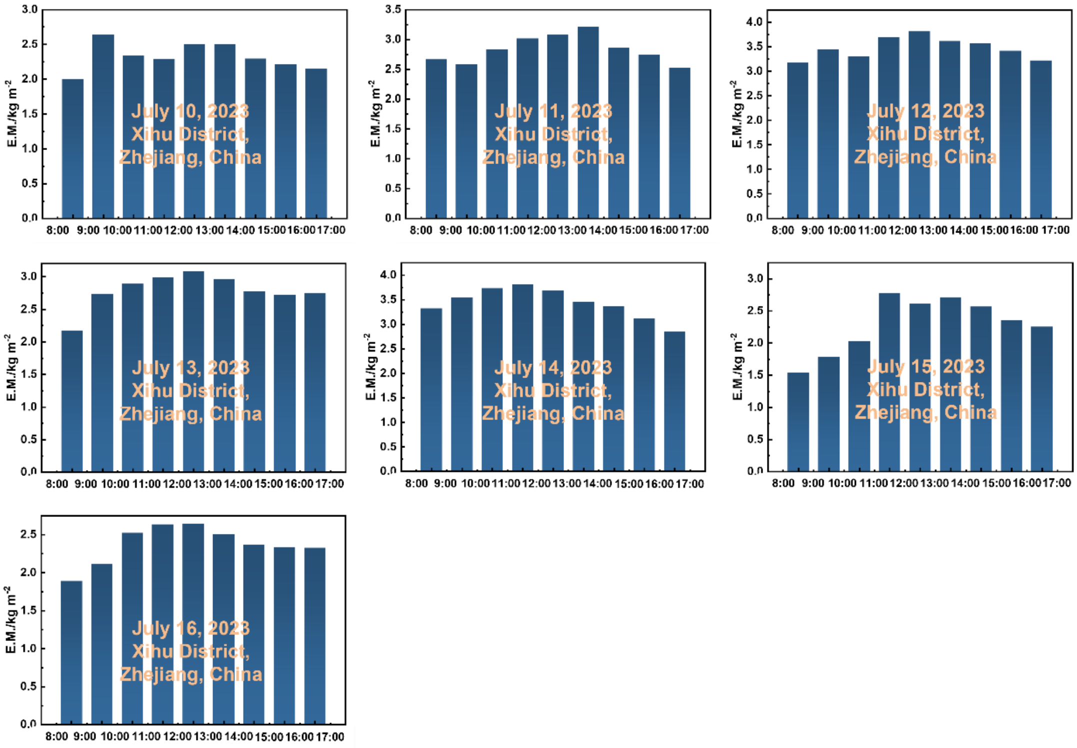

Fig. S26. Details of 3.5 wt% saline evaporation rate for 7-day outdoor cycling evaporation test of FJE (FscP7-P1) solar seawater evaporator.

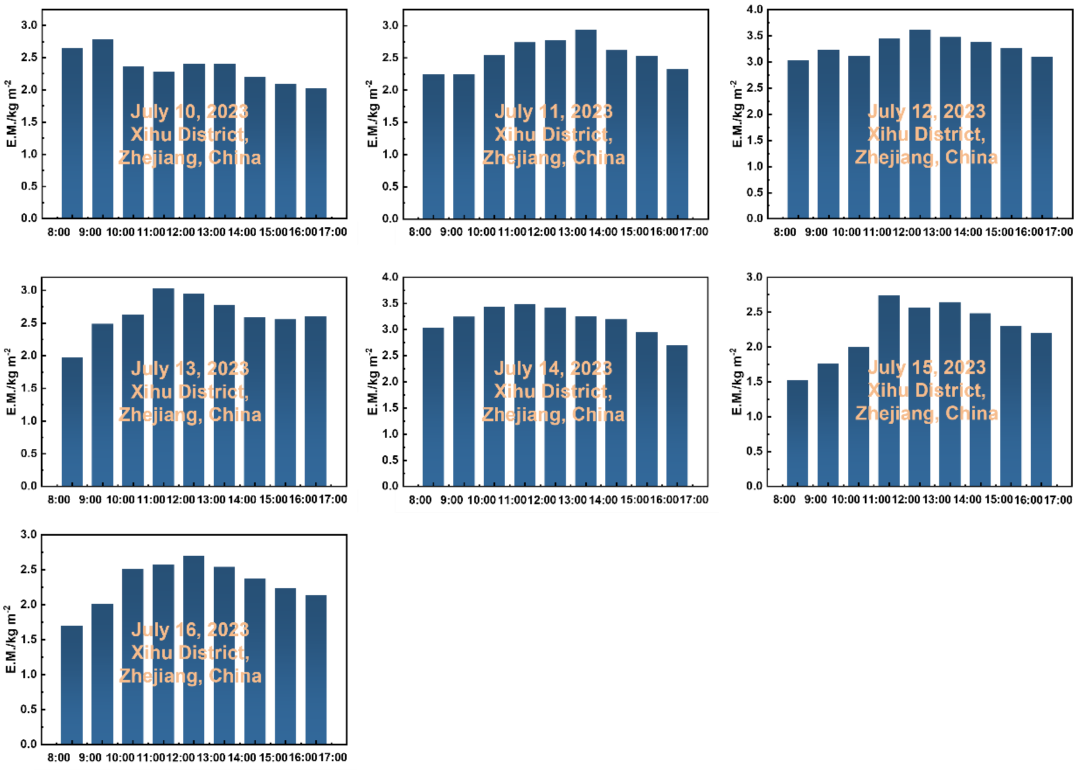

Fig. S27. Details of 20 wt% saline evaporation rate for 7-day Outdoor Cycling Evaporation Test of the FJE (FscP7-P1) Solar Seawater Evaporator.

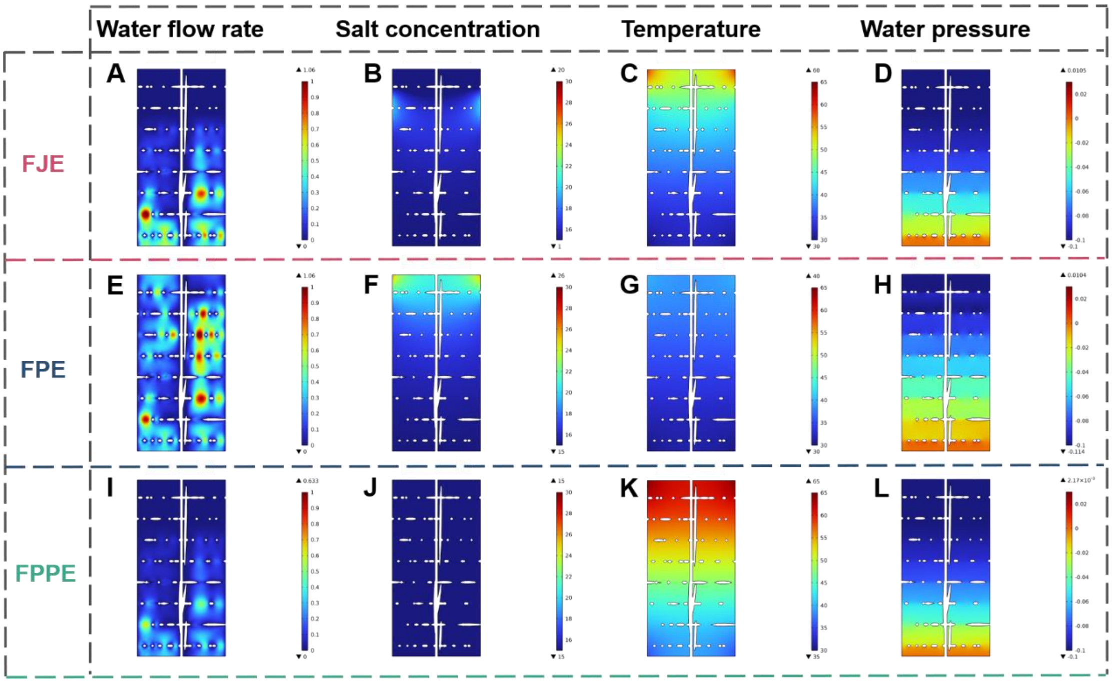

Fig. S28. Simulation of the cross-section of FJE, FPE, and FPPE in aqueous environment to visualize the water flow rate, salt concentration distribution, temperature distribution, and water pressure distribution.

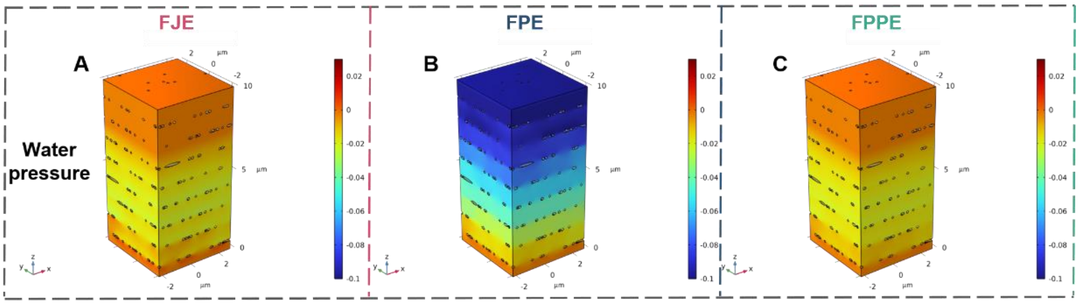

Fig. S29. Simulation of water pressure distribution for (A) FJE, (B) FPE, (C) FPPE

Table S1.

The heat loss of the FPE (FscP7), FJE (FscP7-P1), FPPE (FscP7-P3) during evaporation

Materials	FPE	FJE	FPPE
**Evaporation**	**660.41 W m^{-2}**	**820.98 W m^{-2}**	**598.30 W m^{-2}**
**Radiation**	**38.18 W m^{-2}**	**17.85 W m^{-2}**	**196.85 W m^{-2}**
**Convection**	**28.5 W m^{-2}**	**11.5 W m^{-2}**	**125 W m^{-2}**
**Conduction**	**259.85 W m^{-2}**	**129.62 W m^{-2}**	**64.8 W m^{-2}**

Table S2.

Recent studies on high-efficiency salt-resistant evaporators vs. FJE in Efficiency (%), Salinity (%), evaporation rate (kg·m^{-2}h^{-1}), salt resistance time (h), cost(Usd m^{-2}) and Process (h).

Num	Materials	Efficiency (%)	Salinity (%)	Evaporation rate (kg·m^{-2}h^{-1})	Salt resistance time (h)	Cost (Usd m^{-2})	Process (h)	Ref
1	FJE (Fsc-PPy7-PDMS1)	79.73	20	3.075	18	11.91	3	This work
2	PET/TPU-CNTs	~80	20	2.09	12	226	192	[7]
3	PVA/MNC hydrogel	~70	3.5	2.9	12	83	35	[8]
4	Au@Ag-Pd NSs/Ps microspheres/PVA/diatomite composite film	~80	10	2.84	9	43.29	12.5	[9]
5	HPAN/PVA/CNTs hydrogel	96.1	20	2.33	2.5	902	41	[10]
6	Melamine sponges/TA–APTES complexes/OTS	none	20	2.3	10	2508	38	[11]
7	MXene (Ti3C2) fiber cloth/PVA hydrogel	87.93	15	1.95	5	428	88	[12]
8	PSBMA hydrogel/PPy	81.4	3.5	2.024	10	961	36	[13]
9	PVA/carbon nanofiber 3D sprinting material	99.3	25	2.11	14	101	15	[14]
10	Ni3(HITP)2/Paper	71.1	20	1.6	20	23	2	[15]
11	GFM/CNTs	42	14	1.64	12	44.8	0	[16]
12	PDMS/Bio-graphene/melamine sponge	89.4	3.5	1.42	20	113	9	[17]
13	T-shape C-L-Wood	83.6	3.5	2.43	8	10.5	24	[18]
14	carbonized PVA/PVP hydrogel/PDA-GF/C membrane	78	20	1.4	4	20.4	50	[19]
15	CNTs@SiO2 Nanofibrous Aerogels	~85	20	1.5	10	78	46	[20]
16	FAS/wood	82	20	1.2	8	45	1	[21]
17	CNTs-PVA/EPE foam cylinder	~90	20	1.3	12	16.3	6	[22]
18	Si/PPy-PVA sponge	83.1	26.6	1.36	8	13.86	10	[23]
19	Mxene/Co-MOF foam	91	3.5	1.393	10	14.9	8	[24]

20	Ti3C2Tx MXene/GO/ PANI plastic hybrids	94.7	3.5	3.30	4	208	24	[25]
21	Pt57.5 Ni14.7 Cu5.3 P22.5	94.2	3.5	1.72	1	10.6	2	[26]
22	porous airlaid paper/PPy nanosheet	69.49	3.5	1.38	14	20	0.5	[27]
23	Wood/PPy	83	10	1.33	6	36	12	[28]
24	activated carbon particles/PDMAPS hydrogel	86	10	3.88	20	430	78	[1]
25	Superhydrophilic alumina-based ceramics	none	20	2.3	5	50	18	[29]
26	KGM/PVA/Fe-MOF hydrogel	86	33	3.2	2	14.9	27	[30]
27	CNT/PVA-hydrogel-coated polyester	96.1	4	2.6	16	16.75	7	[31]
28	HNT-cotton 3D evaporator	none	3.5	4.00	9	12.25	3	[32]
29	TiO_2/Ti mesh	94	3.5	1.22	12	20	25	[33]

Table S3.

Details of raw material costs for FJE (FSCP7-P1) solar seawater evaporator. Production and labour costs for FSC are already included in the purchase cost.

Raw materials	Unit Price ($/g)	Dosage (g/m^2)	Total Price ($/m^2)
Flat silk cocoon	0.02	120	2.4
Pyrrole	0.215	33.9	7.29
FeCl3	0.0078	81	0.63
PDMS	0.136	0.7	0.095
Hexane	0.005	132	0.66
PTS	0.043	19.4	0.83
Water	$3.6*10^{-7}$	5000	0.0018
Phosphoric acid	0.0046	1.74	0.008
	Total		11.91

References and Notes

[1] C. Lei, W. Guan, Y. Guo, W. Shi, Y. Wang, K. P. Johnston, G. Yu, *Angew. Chem. Int. Ed.* **2022**, *61* (36), e202208487, https://doi.org/https://doi.org/10.1002/anie.202208487.

[2] X. Liu, F. Chen, Y. Li, H. Jiang, D. D. Mishra, F. Yu, Z. Chen, C. Hu, Y. Chen, L. Qu, W. Zheng, *Adv. Mater.* **2022**, *34* (36), 2203137, https://doi.org/https://doi.org/10.1002/adma.202203137.

[3] Y. Tian, Y. Li, X. Zhang, J. Jia, X. Yang, S. Yang, J. Yu, D. Wu, X. Wang, T. Gao, F. Li, *Adv. Funct. Mater.* **2022**, *32* (33), 2113258, https://doi.org/https://doi.org/10.1002/adfm.202113258.

[4] Z. Liu, B. Wu, B. Zhu, Z. Chen, M. Zhu, X. Liu, *Adv. Funct. Mater.* **2019**, *29* (43), 1905485, https://doi.org/https://doi.org/10.1002/adfm.201905485.

[5] Y. Xu, D. Liu, H. Xiang, S. Ren, Z. Zhu, D. Liu, H. Xu, F. Cui, W. Wang, *J. Membr. Sci.* **2019**, *586*, 222, https://doi.org/https://doi.org/10.1016/j.memsci.2019.05.068.

[6] N. Xu, X. Hu, W. Xu, X. Li, L. Zhou, S. Zhu, J. Zhu, *Adv. Mater.* **2017**, *29* (28), 1606762, https://doi.org/https://doi.org/10.1002/adma.201606762.

[7] H. Liu, B. Chen, Y. Chen, M. Zhou, F. Tian, Y. Li, J. Jiang, W. Zhai, *Adv. Mater.* **2023**, *35* (24), 2301596, https://doi.org/https://doi.org/10.1002/adma.202301596.

[8] H. Zou, X. Meng, X. Zhao, J. Qiu, *Adv. Mater.* **2023**, *35* (5), 2207262, https://doi.org/https://doi.org/10.1002/adma.202207262.

[9] Z. Chen, J. Wang, H. Zhou, Z. Xie, L. Shao, A. Chen, S.-B. Wang, N. Jiang, *Adv. Funct. Mater.* **2023**, *33* (41), 2303656, https://doi.org/https://doi.org/10.1002/adfm.202303656.

[10] W. Ma, T. Lu, W. Cao, R. Xiong, C. Huang, *Adv. Funct. Mater.* **2023**, *33* (23), 2214157, https://doi.org/https://doi.org/10.1002/adfm.202214157.

[11] Z. Wang, J. Gao, J. Zhou, J. Gong, L. Shang, H. Ye, F. He, S. Peng, Z. Lin, Y. Li, F. Caruso, *Adv. Mater.* **2023**, *35* (1), 2209015, https://doi.org/https://doi.org/10.1002/adma.202209015.

[12] L. Li, N. He, B. Jiang, K. Yu, Q. Zhang, H. Zhang, D. Tang, Y. Song, *Adv. Funct. Mater.* **2021**, *31* (43), 2104380, https://doi.org/https://doi.org/10.1002/adfm.202104380.

[13] B. Peng, Q. Lyu, M. Li, S. Du, J. Zhu, L. Zhang, *Adv. Funct. Mater.* **2023**, *33* (18), 2214045, https://doi.org/https://doi.org/10.1002/adfm.202214045.

[14] L. Zhang, Y. Zhang, M. Zou, C. Yu, C. Li, C. Gao, Z. Dong, L. Wu, Y. Song, *Adv. Funct. Mater.* **2023**, *33* (24), 2300318, https://doi.org/https://doi.org/10.1002/adfm.202300318.

[15] Y. Qian, G. Xue, L. Chen, G. Xu, G.-E. Wang, *Adv. Mater.* **2024**, *36* (13), 2310795, https://doi.org/https://doi.org/10.1002/adma.202310795.

[16] K. Yang, T. Pan, S. Dang, Q. Gan, Y. Han, *Nat. Commun.* **2022**, *13* (1), 6653.

[17] M. Wang, Y. Wei, X. Wang, R. Li, S. Zhang, K. Wang, R. Wang, H. Chang, C. Wang, N. Ren, *Nat. Water* **2023**, *1* (8), 716.

[18] H. Liu, R. Jin, S. Duan, Y. Ju, Z. Wang, K. Yang, B. Wang, B. Wang, Y. Yao, F. Chen, *Small* **2021**, *17* (24), 2100969, https://doi.org/https://doi.org/10.1002/smll.202100969.

[19] S. Chaule, J. Hwang, S.-J. Ha, J. Kang, J.-C. Yoon, J.-H. Jang, *Adv. Mater.* **2021**, *33* (38), 2102649, https://doi.org/https://doi.org/10.1002/adma.202102649.

[20] X. Dong, L. Cao, Y. Si, B. Ding, H. Deng, *Adv. Mater.* **2020**, *32* (34), 1908269, https://doi.org/https://doi.org/10.1002/adma.201908269.

[21] X. Chen, S. He, M. M. Falinski, Y. Wang, T. Li, S. Zheng, D. Sun, J. Dai, Y. Bian, X. Zhu, *Energy Environ. Sci.* **2021**, *14* (10), 5347.

[22] Y. Xia, Y. Li, S. Yuan, Y. Kang, M. Jian, Q. Hou, L. Gao, H. Wang, X. Zhang, *J. Mater. Chem. A* **2020**, *8* (32), 16212.

[23] S. Cheng, Z. Yu, Z. Lin, L. Li, Y. Li, Z. Mao, *Chem. Eng. J.* **2020**, *401*, 126108.

[24] X. Fan, Y. Yang, X. Shi, Y. Liu, H. Li, J. Liang, Y. Chen, *Adv. Funct. Mater.* **2020**, *30* (52), 2007110, https://doi.org/https://doi.org/10.1002/adfm.202007110.

[25] X. P. Li, X. Li, H. Li, Y. Zhao, J. Wu, S. Yan, Z. Z. Yu, *Adv. Funct. Mater.* **2022**, *32* (15), 2110636.

[26] J. Fu, Z. Li, X. Li, F. Sun, L. Li, H. Li, J. Zhao, J. Ma, *Nano Energy* **2023**, *106*, 108019.

[27] X. Wang, Q. Liu, S. Wu, B. Xu, H. Xu, *Adv. Mater.* **2019**, *31* (19), 1807716, https://doi.org/https://doi.org/10.1002/adma.201807716.
[28] W. Huang, G. Hu, C. Tian, X. Wang, J. Tu, Y. Cao, K. Zhang, *Sustainable Energy & Fuels* **2019**, *3* (11), 3000.
[29] Z. Wu, D. Sun, C. Shi, S. Chen, S. Tang, Y. Li, C. Yan, Y. Shi, B. Su, *Adv. Funct. Mater.* **2023**, *33* (45), 2304897.
[30] Y. Guo, H. Lu, F. Zhao, X. Zhou, W. Shi, G. Yu, *Adv. Mater.* **2020**, *32* (11), 1907061.
[31] J. Hu, M. M. Pazuki, R. Li, M. Salimi, H. Cai, Y. Peng, Z. Liu, T. Zhao, M. Amidpour, Y. Wei, *Adv. Mater.* **2025**, *37* (14), 2420482.
[32] H. Yu, H. Jin, M. Qiu, Y. Liang, P. Sun, C. Cheng, P. Wu, Y. Wang, X. Wu, D. Chu, *Adv. Mater.* **2024**, *36* (52), 2414045.
[33] M. A. Abdelsalam, M. Sajjad, A. Raza, F. AlMarzooqi, T. Zhang, *Nat. Commun.* **2024**, *15* (1), 874.
